# Targeting the Warburg effect in cancer cells through ENO1 knockdown rescues oxidative phosphorylation and induces growth arrest

**DOI:** 10.18632/oncotarget.6798

**Published:** 2015-12-30

**Authors:** Michela Capello, Sammy Ferri-Borgogno, Chiara Riganti, Michelle Samuel Chattaragada, Moitza Principe, Cecilia Roux, Weidong Zhou, Emanuel F. Petricoin, Paola Cappello, Francesco Novelli

**Affiliations:** ^1^ Department of Molecular Biotechnologies and Health Sciences, University of Turin, Turin 10126, Italy; ^2^ Center for Experimental Research and Medical Studies, University Hospital Città della Salute e della Scienza di Torino, Torino 10126, Italy; ^3^ Department of Oncology, University of Turin, Turin 10126, Italy; ^4^ Center for Applied Proteomics and Molecular Medicine, George Mason University, Manassas, VA 20110, USA; ^5^ Molecular Biotechnology Center, Turin 10126, Italy; ^6^ Immunogenetics and Transplantation Biology Service, University Hospital Città della Salute e della Scienza di Torino, Torino 10126, Italy; ^7^ Department of Clinical Cancer Prevention, The University of Texas MD Anderson Cancer Center, Houston, TX 77030, USA; ^8^ Department of Pathology, The University of Texas MD Anderson Cancer Center, Houston, TX 77030, USA

**Keywords:** alpha-enolase, cancer metabolism, Warburg effect, cellular senescence

## Abstract

In the last 5 years, novel knowledge on tumor metabolism has been revealed with the identification of critical factors that fuel tumors. Alpha-enolase (ENO1) is commonly over-expressed in tumors and is a clinically relevant candidate molecular target for immunotherapy. Here, we silenced ENO1 in human cancer cell lines and evaluated its impact through proteomic, biochemical and functional approaches. ENO1 silencing increased reactive oxygen species that were mainly generated through the sorbitol and NADPH oxidase pathways, as well as autophagy and catabolic pathway adaptations, which together affect cancer cell growth and induce senescence. These findings represent the first comprehensive metabolic analysis following ENO1 silencing. Inhibition of ENO1, either alone, or in combination with other pathways which were perturbed by ENO1 silencing, opens novel avenues for future therapeutic approaches.

## INTRODUCTION

In most solid tumors, the Warburg effect, also known as aerobic glycolysis, causes an increase in total glycolysis both in hypoxic conditions and in the presence of normal oxygen levels [1, 2]. Enhanced proliferation leads to increased anabolic needs and, therefore, cancer cells rewire metabolic pathways to divert nutrients, such as glucose and glutamine, into anabolic pathways to satisfy the demand for cellular building blocks [1]. Thus, to some extent, aerobic glycolysis may give cancer cells an advantage in competing with normal tissues for nutrients [3].

Accumulating evidence shows that the reprogramming of tumor metabolism is controlled by various oncogenic signals [4], such as RAS, AKT, MYC, PI3K, mTOR, together with tumor suppressors, including TP53 and PTEN, which alter metabolism and allow cancer cells to survive and proliferate in the hypoxic and nutrient-deprived tumor microenvironment.

Enolase is a metalloenzyme that catalyzes the dehydration of 2-phospho-D-glycerate to phosphoenolpyruvate in the second half of the glycolytic pathway and is transcriptionally regulated by RAS, MYC and HIF-1α [5–7]. The ENO1 gene can also be translated into a 37 kDa protein, known as c-myc promoter-binding protein (MBP-1), by using an alternative start codon. MBP-1 lacks the first 96 residues of ENO1 and localizes in the nucleus, where it binds to the c-myc P2 promoter and acts as a transcription repressor [8]. ENO1 is upregulated at the mRNA and/or protein level in several tumors including breast, lung, prostate and pancreas and is also expressed on the cell surface of several tumors, where it acts as a plasminogen receptor and contributes to cell invasion, metastasis and inflammatory responses [9–13].

In this study, we have investigated the requirement of ENO1 for maintaining the Warburg effect in cancer cells and we have evaluated whether ENO1 silencing was effective in impairing the growth of cancer cells. ENO1 was silenced in a panel of human cancer cell lines, and we assessed the impact of this silencing through proteomic, biochemical and functional approaches. Unexpectedly, we found that cancer cells respond to ENO1 silencing by activating catabolic adaptations leading to restoration of pyruvate, acetyl-CoA bulk and oxidative phosphorylation. Since ENO1-silenced cancer cells are blocked in cell cycle progression and undergo senescence, ENO1 remains an attractive target for therapeutic intervention in cancer.

## RESULTS

### Proteomics profiling reveals that ENO1 silencing induces oxidative stress in cancer cells

ENO1 was silenced in a human pancreatic cancer cell line, namely CFPAC-1, through a short hairpin RNA targeting ENO1 3â€²UTR (shENO1). After lentiviral infection, ENO1 mRNA and protein levels, as well as enzymatic activity were reduced by around 90% (Supplementary Figure S1).

**Figure 1:**
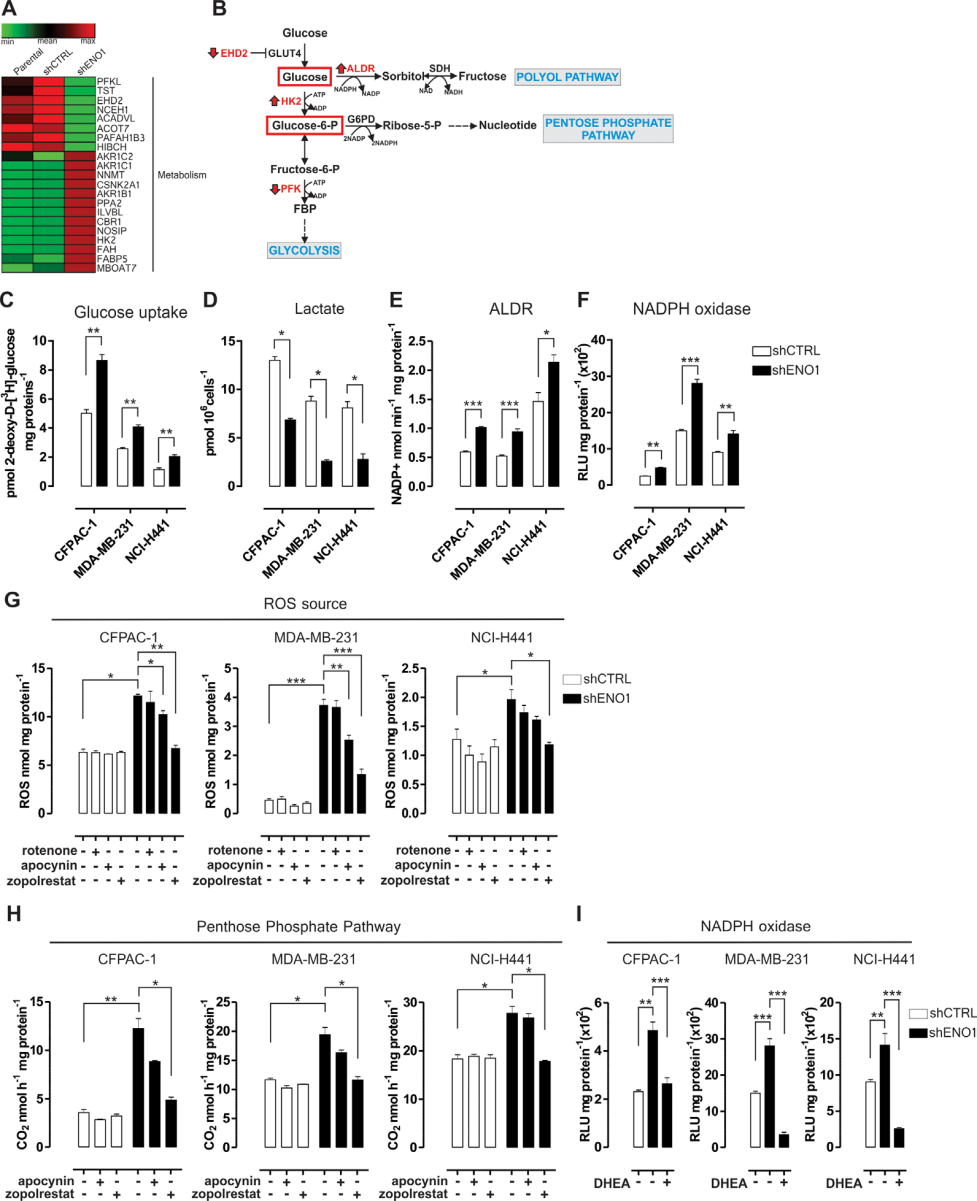
The polyol and pentose phosphate pathways increase the concentration of intracellular reactive oxygen species (ROS) in ENO1-silenced cells **A.** Heat map of proteins differentially expressed in shENO1 compared to shCTRL CFPAC-1 cells (Genesis software). Based on the spectra count label-free quantitation approach, LC-MS/MS analysis identified 13 up-regulated (red) and 8 down-regulated (green) proteins involved in metabolism. Other identified proteins are shown in Supplementary Figure S2A. **B.** Schematic flow chart of the first steps of glycolysis and the branched Polyol Pathway (PP) and the Pentose Phosphate Pathway (PPP). Enzymes that were up- or down-modulated by LC-MS/MS analysis in shENO1 cells compared to shCNTRL cells are shown in red (as indicated by arrows). **C.** Uptake of glucose measured in CFPAC-1, MDA-MB-231 and NCI-H441 cell lines transduced with shCTRL (white bars) or shENO1 (black bars). Uptake was expressed as pmol 2-deoxy-D-[^3^H]-glucose/mg protein. **D.** Analysis of lactate levels in shCTRL and shENO1 CFPAC-1, MDA-MB-231 and NCI-H441 cell lines. **E.**–**F.** Analysis of aldose reductase (ALDR) activity measured by means of the rate of NADPH oxidation (E), and NADPH oxidase activity assessed by the isoluminol-chemiluminescence assay (F) in shCTRL (white bars) and shENO1 (black bars) cell lines. Chemiluminescence was expressed as relative luminescence unit (RLU)/mg protein. **G.**–**H.** Analysis of ROS concentration measured by the DCFDA-AM assay (G) and of [1-^14^C] glucose flux through the PPP, assessed by ^14^CO_2_ release (H) in the presence or absence of inhibitors of the mitochondrial chain (rotenone), NADPH oxidase (apocynin) and ALDR (zopolrestat) in CFPAC-1 (left panels), MDA-MB-231 (middle panels) and NCI-H441 (right panels) cell lines transduced with shCTRL (white bars) or shENO1 (black bars). **I.** Analysis of NADPH oxidase activity, as described above, after selective inhibition of the PPP by dehydroepiandrosterone (DHEA) in CFPAC-1 (left panels), MDA-MB-231 (middle panels) and NCI-H441 (right panels) cell lines transduced with shCTRL (white bars) or shENO1 (black bars). All the graphs illustrate the mean result of three independent experiments ± SEM. *p < 0.05; **p < 0.01; ***p < 0.001 relative to shCTRL.

To systematically identify changes in protein expression in ENO1-silenced cells, a semi-quantitative proteomic analysis was performed. Proteins from CFPAC-1 parental cells, cells infected with a scrambled shRNA (shCTRL) and shENO1 cells were digested using trypsin, and the extracted peptides were identified by liquid chromatography coupled nanospray tandem mass spectrometry (LC-MS/MS) using LTQ-Orbitrap (Supplementary Table S1). The SEQUEST search results were filtered by stringent criteria and yielded 1404 proteins from parental cells, 1489 proteins from shCTRL cells, and 1534 proteins from shENO1 CFPAC-1 cells. Based on the spectra count label-free quantitation approach, 45 up-regulated and 38 down-regulated proteins were identified (Supplementary Table S2–S3). Notably, most of these proteins were involved in cell metabolism, cell adhesion, transport and cell proliferation (Figure 1A, Supplementary Table S2-S3 and Supplementary Figure S2A). To confirm that the observed proteome alterations were not cell-specific, ENO1 was also silenced in two other pancreatic cancer cell lines (PT45 and T3M4) as well as in a breast and a lung tumor cell line (MDA-MB-231 and NCI-H441, respectively) (Supplementary Figure S1B–S1E). Variations in expression of the genes coding for the most relevant proteins (*n* = 19) were evaluated by quantitative real-time PCR analysis in all tumor cell lines (Supplementary Figure S3).

In particular, ENO1-silenced cells showed down-regulation of phosphofructokinase-2 (PFKL), which severely impairs the entry of glucose into glycolysis, and of EH-domain containing 2 (EHD2), which mediates glucose transporter (GLUT4) internalization. Conversely, there was an increased expression of hexokinase-2 (HK2), which catalyzes the first step in glycolysis (Figure 1A–1B, Supplementary Table S2–S3 and Supplementary Figure S3A). Thus, ENO1 silencing led to increased glucose uptake (Figure 1C), which can consequently result in an excess of intracellular glucose being forced into alternative pathways, such as the pentose phosphate pathway (PPP) and the polyol pathway (PP) (Figure 1B). As a result of an impaired glycolytic flux, decreased levels of lactate were observed after ENO1 silencing (Figure 1D).

Several isoforms (AKR1C1, AKR1C2, AKR1B1) of the main enzyme of the PP, aldose reductase (ALDR), were up-regulated in ENO1-silenced cells (Figure 1A–1B, Supplementary Table S2 and Supplementary Figure S3A). In hyperglycemic conditions, PP and ALDR activation induces oxidative stress through the consumption of a strong reducing equivalent, like NADPH [14]. Therefore, ALDR was functionally evaluated and found to be significantly increased in ENO1-silenced cells compared to control cells (Figure 1E and Supplementary Figure S2B). Interestingly NADPH oxidase activity was also increased in ENO1-silenced cells (Figure 1F and Supplementary Figure S2C). Concomitantly, there was a significantly enhanced production of ROS, which was measured by intracellular 5-(and-6)-chloromethyl-2â€²,7â€²-dichorodihydro-fluorescein diacetate (DCFDA) fluorescence and accompanied by a decreased amount of reduced glutathione (GSH) compared to control cells (Figure 1G, Supplementary Figure S2D and data not shown).

To better clarify the ENO1 silencing-dependent origin of ROS, mitochondrial chain, NADPH oxidase and ALDR were inhibited by means of rotenone, apocynin and zopolrestat, respectively. The latter two reduced ROS levels in ENO1-silenced cells, while rotenone did not (Figure 1G and Supplementary Figure S2D), suggesting that ALDR and – to a lesser extent – NADPH oxidase, but not the mitochondria electron flux, were the major sources of ROS production in ENO1-silenced cells. The flux of [1-^14^C] glucose via the PPP was also increased in ENO1-silenced cells, as assessed by measuring ^14^CO_2_ release (Figure 1H and Supplementary Figure S2E). Zopolrestat, but not apocynin, reduced ^14^CO_2_ release, suggesting that the increase in ALDR activity led to a decrease in NADPH and, in turn, to activation of the PPP in ENO1-silenced cells. Finally, inhibition of the PPP by dehydroepiandrosterone (DHEA) (Supplementary Figure S2F) induced a drop in the activity of NADPH oxidase (Figure 1I), suggesting that the greater NADPH oxidase activity was supported by the enhanced PPP flux. The increase in ALDR and NADPH oxidase activity finally contributed to the higher ROS levels of ENO1-silenced cells.

### ENO1 silencing enhances oxidative stress induced-autophagy, fatty acid oxidation and amino acid catabolism

**Figure 2:**
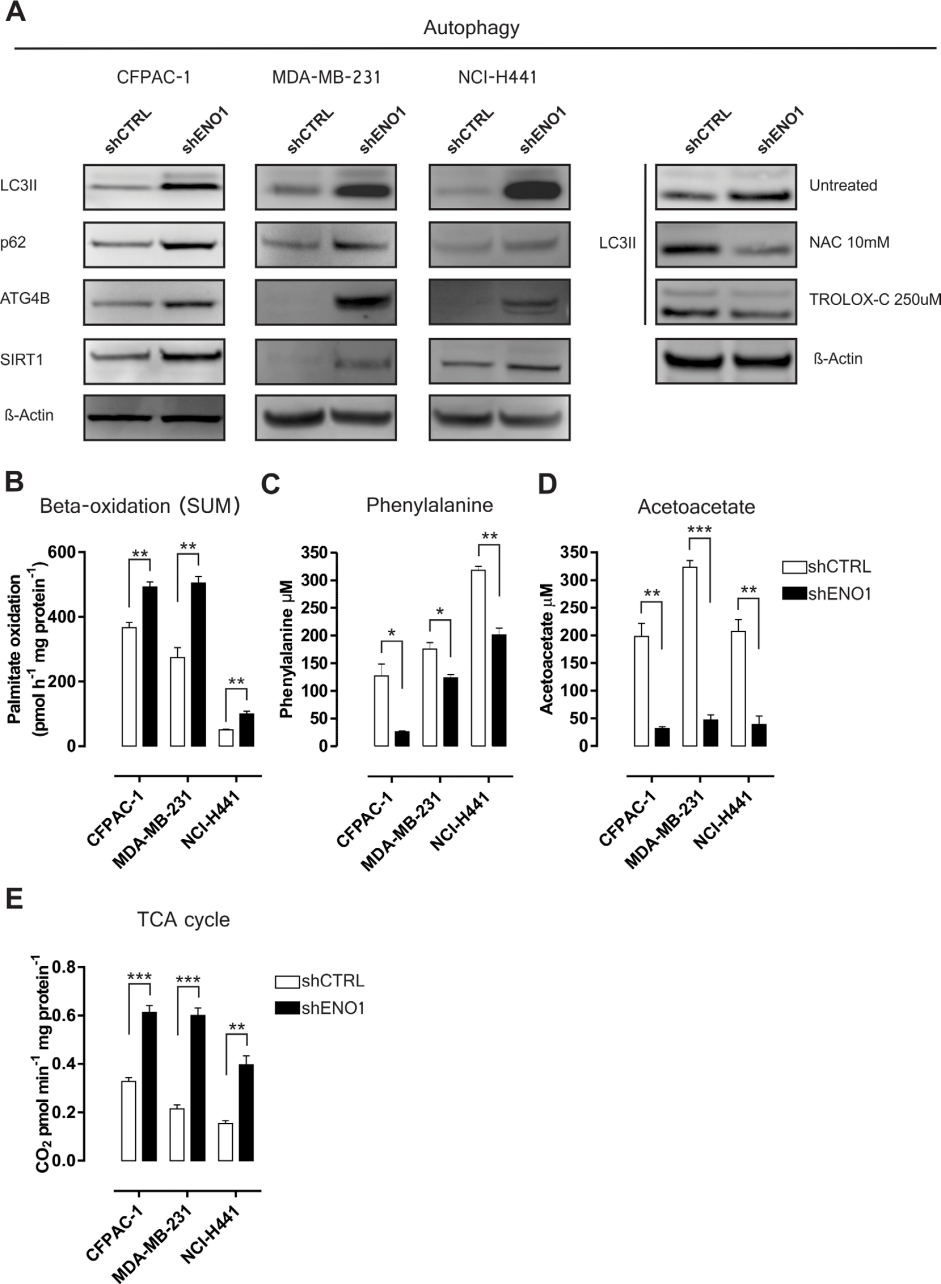
ENO1 silencing enhances catabolic pathway adaptations **A.** Autophagy markers and SIRT-1 Western blot expression analysis in shCTRL and shENO1 CFPAC-1, MDA-MB-231 and NCI-H441 cells. Right panel: effects of the antioxidant agents NAC and TROLOX-C on LC3-II expression in CFPAC-1 cells by Western blot analysis. β-actin was used as a loading control; one representative of three independent experiments is shown **B.** Oxidation of palmitic acid in CFPAC-1, MDA-MB-231 and NCI-H441 cell lines transduced with shCTRL (white bars) or shENO1 (black bars). Cells were exposed to [1-^14^C] palmitic acid and total palmitate oxidation (sum of ^14^C-acid soluble metabolites and ^14^CO_2_ production) was measured. **C.**–**D.** Analysis of phenylalanine (C) and acetoacetate (D) concentration in shCTRL and shENO1 CFPAC-1, MDA-MB-231 and NCI-H441 cell lines. **E.** The TCA cycle rate was evaluated measuring CO_2_ emission after radiolabeling cells with [1-^14^C] acetylcoenzyme A in CFPAC-1, MDA-MB-231 and NCI-H441 cell lines transduced with shCTRL (white bars) or shENO1 (black bars). TCA cycle activity is expressed as pmol CO_2_/min/mg protein. All graphs illustrate the mean result of three independent experiments ± SEM. *p < 0.05; **p < 0.01;***p < 0.001 relative to shCTRL.

The two most up-regulated proteins due to ENO1 silencing were sequestosome 1 (SQSTM1/p62) and nicotinamide N-methyl transferase (NNMT) (Supplementary Table S2 and Supplementary Figure S3B). These enzymes are involved in oxidative stress- and sirtuin-induced autophagy, respectively [15–19]. Notably, ENO1-silenced cells showed an increased expression of autophagic markers such as LC3-II, p62 and ATG4B, together with Sirtuin-1 (Sirt-1) (Figure 2A). To determine whether the induction of autophagy after ENO1 silencing was related to the increased amount of ROS, the effect of antioxidants was evaluated. A 7-day treatment with both N-acetyl-cysteine (NAC) and 6-hydroxy-2,5,7,8-tetramethylchromane-2-carboxylic acid (TROLOX-C) reduced the amount of LC3-II marker in ENO1-silenced cells (Figure 2A, right panel).

In ENO1-silenced cells, mass spectrometry showed an altered expression of neutral cholesterol ester hydrolase 1 (NCEH1), acyl-CoA dehydrogenase very long chain (ACADVL), acyl-CoA thioesterase 7 (ACOT7), platelet-activating factor acetylhydrolase 1b catalytic subunit 3 (PAFAH1B3), fatty acid binding protein 5 (FABP5) and membrane bound O-acyltransferase domain-containing 7 (MBOAT7) (Figure 1A, Supplementary Table S2–S3 and Supplementary Figure S3B), which suggests enhanced fatty acid oxidation. To confirm this hypothesis, the oxidation of [1-^14^C] palmitic acid was determined by measuring ^14^C-acid soluble metabolites (ASM) and by the trapping of [1-^14^C] palmitic acid-derived ^14^CO_2_. Palmitate oxidation (sum of ASM and CO_2_) was significantly increased in ENO1-silenced cells compared to control cells (Figure 2B and Supplementary Figure S4A–S4C), confirming that ENO1 silencing perturbed lipid metabolism and promoted beta oxidation with the probable restoration of acetyl-CoA bulk.

Fumarylacetoacetase (FAH), an enzyme involved in tyrosine catabolism, which catalyzes the hydrolysis of 4-fumarylacetoacetate into fumarate and acetoacetate, was up-regulated after ENO1 silencing (Figure 1A, Supplementary Table S2 and Supplementary Figure S3B). In addition, as tyrosine is synthesized from phenylalanine, quantification of phenylalanine and acetoacetate levels was carried out, and a decrease in concentration of both molecules was observed after ENO1 silencing (Figure 2C–2D, Supplementary Figure S4D-E and S4G). The increased rate of phenylalanine and lipid catabolism may feed the tricarboxylic acid (TCA) cycle, which was indeed increased in ENO1-silenced cells (Figure 2E and Supplementary Figure S4F), by anaplerotic reactions (Supplementary Figure S4G).

### ENO1 silencing induces a drop in nucleotide base synthesis and promotes oxidative phosphorylation

**Figure 3:**
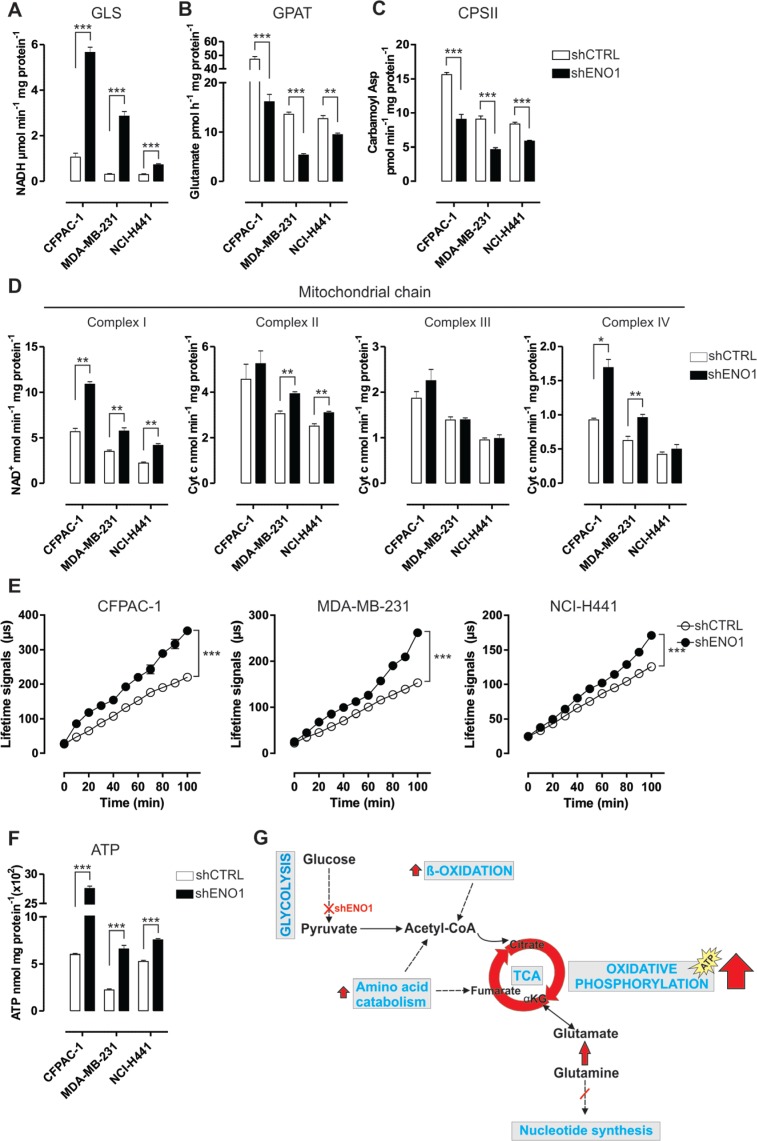
ENO1 silencing induces a decrease in nucleotide base synthesis and promotes oxidative phosphorylation **A.**–**C.** Analysis of glutaminase (GLS) (A), glutamine amidophosphoribosyltransferase (GPAT) (B) and carbamoyl phosphate synthetase II (CPSII) (C) activity in CFPAC-1, MDA-MB-231 and NCI-H441 cell lines transduced with shCTRL (white bars) or shENO1 (black bars). GLS activity is expressed as μmol NADH/min/mg protein. GPAT activity is an index of the *de novo* synthesis of purine nucleotides and is expressed as pmol glutamate/h/mg protein. CPSII activity is an index of the *de novo* synthesis of pyrimidine nucleotides and is expressed as pmol carbamoyl aspartate/min/mg protein. **D.** Analysis of the activity of mitochondrial respiratory chain complex I and complexes II-IV in CFPAC-1, MDA-MB-231 and NCI-H441 cell lines transduced with shCTRL (white bars) or shENO1 (black bars), expressed as nmol NAD^+^/min/mg mitochondrial protein for complex I, nmol Cyt c reduced/min/mg mitochondrial protein for complexes II-III, nmol Cyt c oxidized/min/mg mitochondrial protein for complex IV. **E.** Analysis of oxygen consumption in CFPAC-1, MDA-MB-231 and NCI-H441 cell lines transduced with shCTRL (white dots) or shENO1 (black dots). Results were expressed as the lifetime signal of the fluorescent probe MitoXpress provided in the kit versus the assay duration (μs). Curves were compared by two-way ANOVA, ***p < 0.001. **F.** Analysis of ATP production in CFPAC-1, MDA-MB-231 and NCI-H441 cell lines transduced with shCTRL (white bars) or shENO1 (black bars). All graphs illustrate the mean result of three independent experiments ± SEM. *p < 0.05; **p < 0.01;***p < 0.001 relative to shCTRL. **G.** Cartoon illustrating the catabolic pathway adaptations induced by ENO1 silencing. ENO1 silencing promotes fatty acid beta oxidation, which restores acetyl-CoA bulk and increases the TCA anaplerotic reactions derived from phenylalanine catabolism. These events, together with the increased entry of glutamine-derived metabolites into the TCA cycle, induce a decrease in nucleotide base synthesis and promote oxidative phosphorylation.

Many cancer cells and, in particular, pancreatic cancer cells, show specific addiction to glutamine, which provides a source of carbon to fuel the TCA cycle, and of nitrogen for nucleotide biosynthesis, as well as NADPH for redox maintenance [20]. ENO1-silenced cells showed an increased activity of glutaminase (GLS), which converts glutamine into glutamate to fuel the TCA cycle, and a concomitant reduction of both glutamine amidophosphoribosyltransferase (GPAT) and carbamoyl phosphate synthetase II (CPSII) activity, which hydrolyze glutamine for *de novo* synthesis of purine and pyrimidine nucleotides, respectively (Figure 3A–3C and Supplementary Figure S5A–S5C). These results suggest that glutamine metabolites were rapidly consumed in the TCA cycle rather than for nucleic acid synthesis. Up-regulation of the electron flux in the mitochondrial chain, especially through complexes I and II was observed in all cell lines after ENO1 silencing, along with increased oxygen consumption (Figure 3D–3E and Supplementary Figure S5D). These observations suggest that ENO1-silenced cells tend to consume more O_2_ to sustain oxidative phosphorylation.

Consequently, there was a strong increase in ATP synthesis (Figure 3F and Supplementary Figure S5E). Overall, ENO1 silencing switched the typical aerobic glycolysis of cancer cells towards oxidative phosphorylation (Figure 3G).

In accordance with these metabolic adaptations, the mRNA levels of liver kinase B1 (LKB1), 5â€² AMP-activated protein kinase (AMPK1α) and peroxisome proliferator-activated receptor gamma coactivator 1-alpha (PGC1α), which are mediators of catabolic metabolism and mitochondria biogenesis, were increased after ENO1 silencing (Supplementary Figure S5F).

To confirm that the metabolic adaptations observed after ENO1 silencing were not dependent on the exposure of the cells to high glucose concentrations, we mimicked physiological conditions by culturing cancer cells in low glucose medium. There were no differences in ALDR, GLS, GPAT, CPSII or mitochondrial respiratory chain activity, as well as no change in ATP production in shCTRL or shENO1 CFPAC-1 cells after 24 h culture in low (1 g/L) or high (4.5 g/L) glucose media (Supplementary Figure S6A–S6F).

### ENO1 silencing impairs cancer cell growth both *in vitro* and *in vivo*

**Figure 4:**
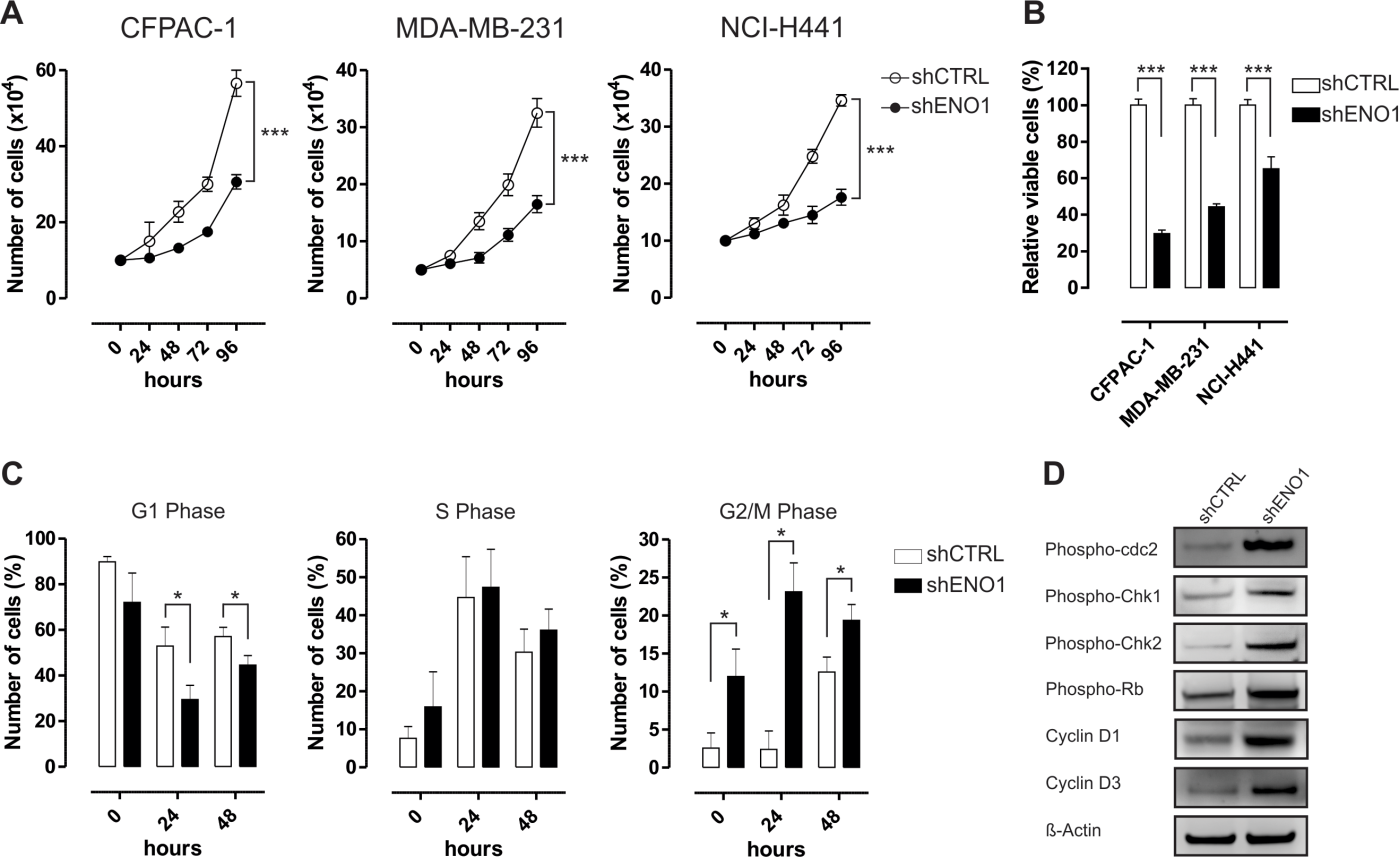
ENO1 silencing impairs cancer cell growth **A.** Cell proliferation analysis of CFPAC-1 (left panel), MDA-MB-231 (middle panel) and NCI-H441 (right panel) cell lines growth after transduction with shCTRL (white circles) or shENO1 (black circles). Cells were starved and counted every 24 h after serum replenishment. Curves were compared by two-way ANOVA, ***p < 0.001. **B.** Cell survival assessed by MTT assay. Cells transduced with shCTRL (white bars) or shENO1 (black bars) were starved and MTT solution was added 48 h after serum replenishment. OD values were measured at 570 nm. **C.** Flow cytometry cell cycle analysis of serum-starved shCTRL and shENO1 CFPAC-1 cells at the indicated time points after serum replenishment. Graphs represent the percentage of cells at each phase. **D.** Western blot analysis of shCTRL and shENO1 CFPAC-1 cells with the indicated antibodies. Cells were serum starved and lysed at 24 h after serum replenishment. β-actin was used as a loading control. One representative out of three independent experiments is shown. Results are reported in the graphs as means of three independent experiments ± SEM. *p < 0.05, **p < 0.01, ***p < 0.001 relative to shCTRL.

ENO1 silencing decreased cancer cell growth, survival and clonogenic capability *in vitro*, as evaluated by cell growth curve, MTT assay, and colony formation assay, respectively (Figure 4A–4B and Supplementary Figure S7A–S7C), without any evidence of apoptosis (data not shown). The cell-cycle profile analysis after 24 h serum deprivation revealed a significant increase in the number of ENO1-silenced cells in G2/M phase, a concomitant decrease of cells in G1 phase and no difference in the number of cells in S phase (Figure 4C and Supplementary Figure S7D–S7E). In CFPAC-1 cell line the blockade of ENO1-silenced cells in G2/M phase correlated with an increase in: i) phosphorylation of the catalytic subunit of the M-phase promoting factor cdc2; ii) phosphorylation of the kinases Chk1 and Chk2, mediators of the G2/M DNA damage checkpoint, iii) expression of cyclin D1 and phosphorylation of Rb, which promote progression through G1/S phases; iv) expression of cyclin D3, a key factor in the progression from G2 to M phase (Figure 4D). Conversely, there was a decrease in expression of the negative regulator of the cyclin D/CDK complex p18 (INK4C) after ENO1 silencing (Figure 4D).

**Figure 5:**
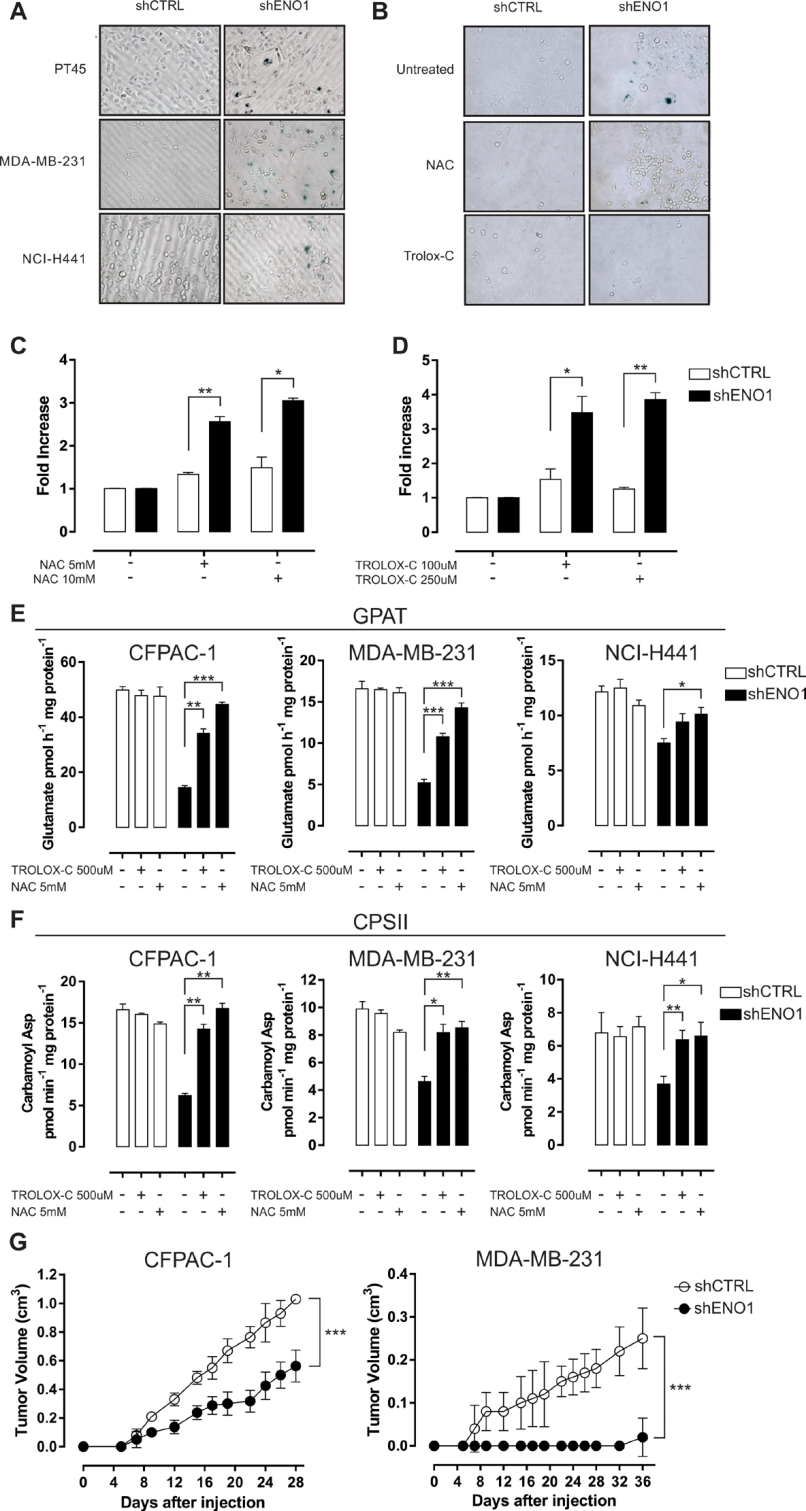
ENO1 silencing induces cellular senescence **A.** Senescence-associated β-galactosidase staining. Senescent PT45, MDA-MB-231 and NCI-H441 cells were colored blue upon X-gal staining at pH 6. One representative out of three independent experiments is shown. **B.**–**D.** Effects of antioxidants on shENO1 cells. After a 7-day treatment with anti-oxidant N-acetyl-cysteine (NAC) or 6-Hydroxy-2,5,7,8-tetramethylchromane-2-carboxylic acid (TROLOX-C), PT45 cells were stained with X-gal at pH 6 to detect the presence of senescent cells. One representative out of three independent experiments is shown (B). Cell growth was assessed after treatment with NAC (C) or TROLOX-C (D). Bars represent fold-increase in number of cells relative to untreated shCTRL (white bars) or shENO1 (black bars) cells. **E.**–**F.** Analysis of glutamine amidophosphoribosyltransferase (GPAT) (E) and carbamoyl phosphate synthetase II (CPSII) (F) activity in CFPAC-1, MDA-MB-231 and NCI-H441 cell lines after treatment with NAC or TROLOX-C. GPAT activity is expressed as pmol glutamate/h/mg protein. CPSII activity is expressed as pmol carbamoyl aspartate/min/mg. Results are means of three independent experiments ± SEM. *p < 0.05, **p < 0.01, ***p < 0.001 relative to shCTRL. **G.** *In vivo* growth of shCTRL (white circles) or shENO1 (black circles) CFPAC-1 (left panel) or MDA-MB-231 (right panel) injected s.c. in SCID-beige mice. The graph represents the mean tumor volume (*n* = 5 mice/group). Curves were compared by two-way ANOVA, ***p < 0.001.

Notably, ENO1-silenced cells showed characteristic morphological changes, such as enlargement and flattening, which were indicative of cellular senescence, confirmed by β-galactosidase staining (Figure 5A and Supplementary Figure S7F). To confirm whether the delay in proliferation after ENO1 silencing was due to an increased amount of ROS, the effect of antioxidant agents was evaluated. A 7-day treatment with either NAC or TROLOX-C reduced the number of senescent ENO1-silenced cells (Figure 5B), and simultaneously increased their proliferative ability (Figure 5C–5D). ROS blunting indeed rescued GPAT and CPSII activity in ENO1-silenced cells (Figure 5E–5F), while ALDR and mitochondrial respiratory chain activity, beta-oxidation, and TCA cycle rate were not affected by antioxidant treatment (Supplementary Figures S8A–S8B and S9A–S9D).

For *in vivo* investigations, ENO1-silenced or control CFPAC-1 and MDA-MB-231 cells were subcutaneously (s.c.) injected into the flanks of SCID-beige mice, and tumor growth was monitored. ENO1 silencing resulted in a profound reduction of tumor volume compared to that observed when control cells were injected (Figure 5G).

### ENO1 targeting shows translational relevance

The increased levels of both ROS and GLS activity after ENO1 silencing prompted us to investigate whether ENO1-deficient cancer cells could be sensitized to the inhibition of anabolic glutamine metabolism. To test this hypothesis, we inhibited glutamine metabolism using the GLS inhibitor BPTES (bis-2-(5phenylacetamido-1,2,4-thiadiazol-2-yl) ethyl sulfide), and examined its synergism with ENO1 silencing. GLS inhibition significantly decreased CFPAC-1, but not MDA-MB-231 or NCI-H441 cell survival, suggesting that pancreatic cancer cells are considerably more sensitive to GLS inhibition when ENO1 function is impaired (Supplementary Figure S10A).

**Figure 6:**
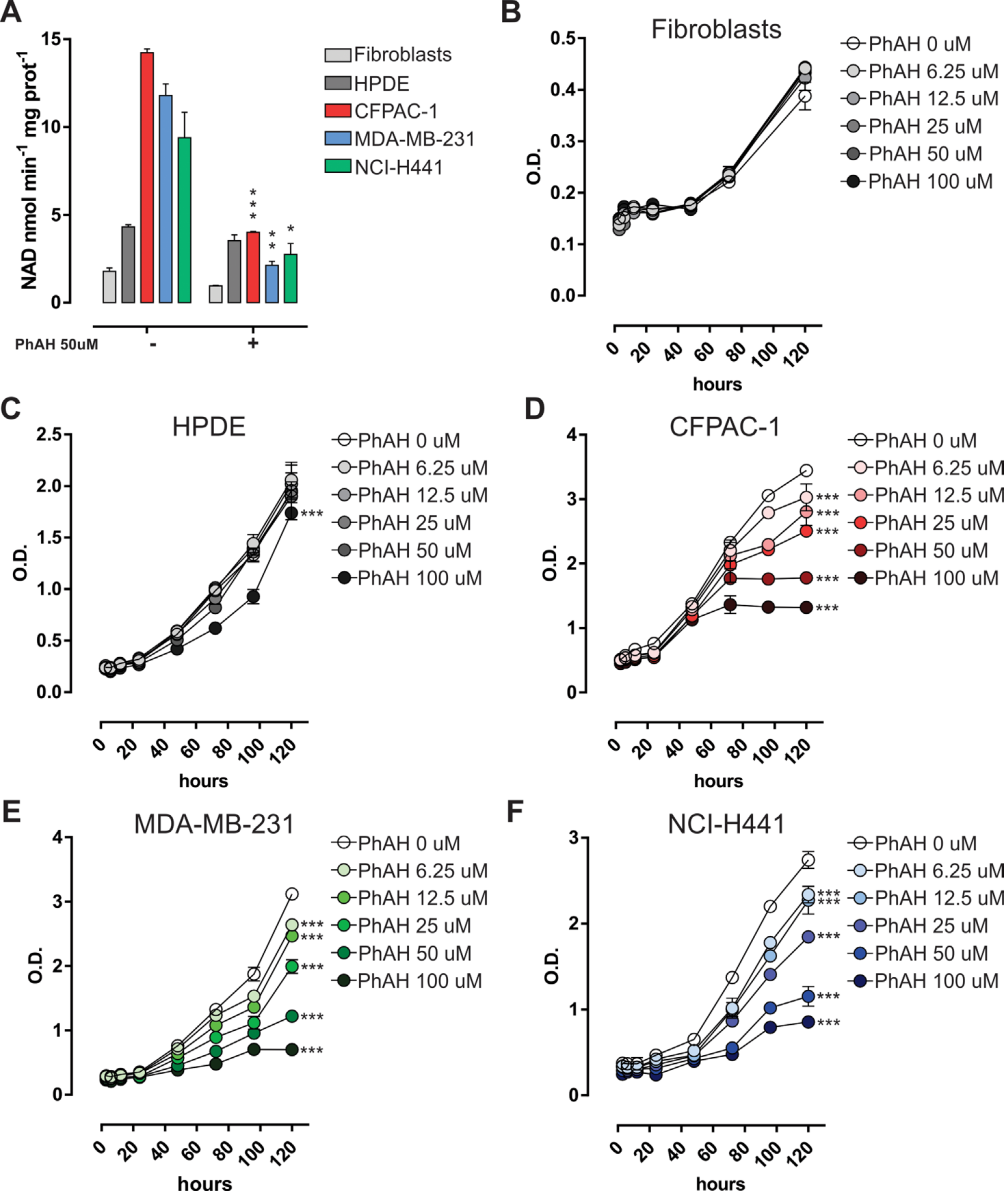
Sensitivity of cancer cells to the pan-enolase inhibitor PhAH **A.** Analysis of enolase activity measured as the rate of NADH oxidation in normal human fibroblasts, HPDE, CFPAC-1, MDA-MB-231 and NCI-H441 cell line protein extracts treated for 6 h with 50 μM PhAH. **B.**–**F.** Time- and dose-dependent effect of enolase pharmacological inhibitor (PhAH) on cell survival assessed by MTT assay. Cells were starved and MTT solution was added every 24 h after serum replenishment and PhAH boost. OD values were measured at 570 nm. Curves were compared by two-way ANOVA, ***p < 0.001 relative to untreated.

The effect of the pharmacological inhibitor of enolase, namely phosphonoacetohydroxamate (PhAH), a transition-state analogue (21), on cell viability, was also evaluated. PhAH inhibited enolase enzymatic activity in cancer cell lines but not in normal (non-transformed) cells, where enolase activity was significantly lower (Figure 6A). PhAH significantly reduced proliferation of CFPAC-1, MDA-MB-231, and NCI-H441 cells, but not of normal human fibroblasts or human pancreatic ductal epithelial (HPDE) cells, in a dose-dependent manner, as assessed by MTT assay (Figure 6B–6F). Enolase enzymatic activity was inversely correlated with the percentage of viable cells relative to untreated control cells after 120 h treatment with 50 μM (*r* = −0.888, *p* = 0.0443; Pearson Correlation) and 100 μM (*r* = −0.900, *p* = 0.0376; Pearson Correlation) PhAH, whereby the cell lines with greater enolase activity were more sensitive to PhAH treatment. Moreover, enolase enzymatic activity after 6 h treatment with different doses of PhAH (range 6.25–100 μM) and the percentage of viable cells relative to untreated control cells after treatment with the same doses of PhAH were directly correlated in CFPAC-1 cells at different time-points (range 3 h–120 h) (Supplementary Figure S10B–S10C), further confirming a specific dose-dependent effect on cell viability of enolase enzymatic activity inhibition.

## DISCUSSION

Cancer cells show increased aerobic glycolysis and enhanced lactate production compared to healthy cells, a phenomenon known as the Warburg effect [2]. Furthermore, tumor tissue accumulates a greater amount of glucose than healthy tissue, as it requires more glucose as a carbon source for anabolic reactions. This biological adaptation to metabolic changes owing to mitochondrial dysfunction, hypoxia and oncogenic signals, results in glycolysis being favored by malignant cells, as opposed to the more “energetic” oxidative phosphorylation. Attenuation or inhibition of glycolysis has been shown to be useful for preventing cancer development, demonstrating that glycolysis is essential for proliferation, invasion and metastasis of cancer [22].

The multifunctional glycolytic enzyme α-enolase (ENO1) has been shown to be commonly over-expressed in tumors [9, 12, 23], and is thus a promising and clinically-relevant molecular target for immunotherapeutic approaches, particularly in pancreatic cancer [10, 13, 24]. In this study, we explored the hypothesis that ENO1 is one of the leading regulators of the Warburg effect and thus plays a major role in carcinogenesis and tumor maintenance. We found that ENO1 silencing in tumor cells affected the expression of all enolase isoforms, confirming that the inhibition of enolases decreases proliferation [25] and also affects *in vivo* tumor growth.

The most important observation of our work is that, surprisingly, ENO1-silenced cells were able to resist glycolytic shutdown by means of the rescue of oxidative phosphorylation. Mass spectrometry analysis revealed an up-regulation in HK2 expression and a down-regulation in PFKL expression after ENO1 silencing. In addition, EHD2, which mediates glucose transporter internalization, was down-modulated. In the absence of ENO1, the decrease in lactate production and increase in ATP demand promote glucose uptake and eventually lead to the accumulation of intermediate glycolytic metabolites. Therefore, the excess of intracellular glucose must be redistributed towards alternative pathways, such as the polyol pathway (PP) and the pentose phosphate pathway (PPP) to support cell growth and survival. The PP metabolizes glucose into sorbitol and NADH and is typically activated in hyperglycemic states. The PP induces oxidative stress through the consumption of a strong reducing equivalent like NADPH [14]. Moreover, PP activation results in a decrease of reduced NADPH and oxidized NAD^+^, which are necessary cofactors in redox reactions, leading to decreased synthesis of reduced glutathione. The PPP plays a critical role in regulating cancer cell growth by supplying cells with both ribose-5-phosphate and NADPH for detoxification of intracellular ROS [3]. In ENO1-silenced cells, the activities of the PP and the PPP were increased and associated with an enhanced activity of ALDR, the main enzyme of the PP and an important contributor to cell oxidative stress. The increased activity of ALDR resulted in decreased expression of NADPH and, in turn, activation of the PPP, which promotes NADPH oxidase activation by restoring NADPH bulk. As demonstrated by the experiments with the PPP inhibitor DHEA, NADPH oxidase hyperactivation was a consequence of the increased PPP flux and further contributed to the synthesis of superoxide. ROS are responsible for functional changes observed in ENO1-silenced cells, such as growth arrest and senescence, as demonstrated by treatment with antioxidants or inhibitors of cellular oxidant scavengers [26]. Indeed, we observed the rescue of proliferation and senescence upon administration of antioxidants, and we therefore went on to investigate whether inhibiting antioxidant pathways could lead to cell death. The effectiveness of PP or PPP inhibitors in controlling cell proliferation, survival and senescence have been demonstrated [27–30]. In our model, concomitant inhibition of ENO1 and the main enzymes of the PP or PPP, such as ALDR and glucose-6-phosphate dehydrogenase (G6PD), either by shRNA or chemical inhibitors, did not enhance the effects of ENO1 silencing on cell proliferation (data not shown). It is likely that the simultaneous inhibition of ENO1 and the PPP enables metabolic compensation for the synthesis of R5P through the alternative non-inhibited branch of the PPP.

Effective removal of oxidative-damaged proteins both during glycolysis and oxidative phosphorylation is essential for maintaining a clear distinction between controlled ROS for cell signaling and uncontrolled redox-dependent pathogenesis. A major pathway responsible for removing these damaged macromolecules and organelles is the autophagy–lysosomal pathway. Autophagy has been shown to be regulated by numerous factors of glucose metabolism and can be activated by mitochondrial dysfunction [31, 32]. In our study, ENO1-silenced cells showed an increased expression of proteins involved in both oxidative stress- and sirtuin-induced autophagy. Moreover, antioxidant treatment reduced LC3-II levels, thus correlating ROS production with the induction of autophagy in ENO1-silenced cells. Notably, autophagy is also the most important stress response for cells in adapting to nutrient starvation, and is a well-known effector of senescence [33]. Interestingly, even though ENO1-deficient cells show an up-regulation of autophagy, they do not rely on this catabolic process for their survival, as ENO1-silenced cells do not show increased sensitivity to the autophagy inhibitors Bafilomycin A1 and Hydroxychloroquine (data not shown).

We have observed that ENO1 silencing also promotes catabolic pathway adaptations, restores acetyl-CoA bulk through enhanced β-oxidation and fuels TCA by the anaplerotic reactions of tyrosine and glutamine catabolism. The addiction of cancer cells to glutamine, which provides the carbon source to fuel the TCA cycle, NADPH for redox maintenance and nitrogen for nucleotide biosynthesis, is a well-known phenomenon [20, 34–37]. After ENO1 silencing, we observed an increased activity of glutaminase, which provides glutamate for conversion into α-ketoglutarate, together with a reduced activity of both GPAT and CPSII, enzymes that hydrolyze glutamine for the *de novo* synthesis of purine and pyrimidine nucleotides, respectively. Our results therefore suggest that catabolic adaptations in ENO-1 silenced cells increase TCA cycle activity, induce a decline in nucleotide base synthesis and promote cellular senescence, which can be rescued by ROS blunting [38]. Interestingly, GLS inhibition significantly decreased CFPAC-1 survival suggesting that pancreatic cancer cells, known to have specific addiction to glutamine [20], are markedly more sensitive to GLS inhibition when ENO1 function is impaired. However, the co-lethality paradigm applies specifically to pancreatic cancer cells, as simultaneous inhibition of ENO1 and GLS did not synergize in inhibiting MDA-MB-231 or NCI-H441 breast and lung cancer cell survival.

Warburg considered that aerobic glycolysis in cancer cells was due to an irreversible impairment of mitochondrial function. This view has been challenged by the observation of the intact function of mitochondrial oxidative phosphorylation in many cancers [39–41]. Indeed, in ENO1-silenced cells, we showed that mitochondrial electron flux actually increased oxygen consumption and promoted ATP synthesis, restoring oxidative phosphorylation. In addition to this catabolic adaptation, ENO1-silenced cells underwent G2/M phase cell cycle arrest and cellular senescence.

In conclusion, our study has provided comprehensive proteomics, biochemical and functional data to demonstrate that ENO1 is a master regulator of tumor metabolism. The effect of ENO1 silencing opens new possibilities for cancer treatment, based on a combination between ENO1 targeting and therapies that are able to increase oxidative and metabolic stress, such as chemotherapy and radiation, or glutaminase and oxidative phosphorylation inhibitors, respectively. Of particular significance, we showed that a pan-enolase inhibitor, PhAH, was able to induce a remarkable decrease of cancer cell proliferation *in vitro*, supporting the translationability of ENO1 inhibition.

## MATERIALS AND METHODS

### Cell culture and viral transduction

The cell lines used in this study were: CFPAC-1 (ECACC ref no. 91112501), T3M4, HPDE and PT45 (kindly provided by Dr. P. Nisticò, Regina Elena National Cancer Institute, Rome, Italy), MDA-MB-231 (kindly provided by Dr. P. Michieli, University of Turin, Turin, Italy), NCI-H441 (kindly provided by Dr. R. Chiarle University of Turin, Turin, Italy) and normal human fibroblasts (kindly provided by Dr. C. Castagnoli, Burn Centre of Turin, Turin, Italy). All cell lines were maintained in DMEM (Lonza) supplemented with 10% FBS (Lonza), 2 mM L-Glutamine (Gibco) and 50 mg/ml of gentamicin (Sigma-Aldrich). All cell lines were routinely tested for mycoplasma contamination. Lentiviral infections were performed as described in the Supplementary Materials and Methods.

### Quantitative RT–PCR and Western blot analysis

Quantitative RT-PCR and Western blot analysis are described in the Supplementary Materials and Methods.

### Tandem mass spectrometry analysis

The preparation and measurement of proteins by highly sensitive reversed-phase liquid chromatography coupled nanospray tandem mass spectrometry (LC-MS/MS) are described in the Supplementary Materials and Methods.

### ROS measurement

Cells were rinsed with PBS, detached by gentle scraping, resuspended in 0.5 mL PBS, and loaded with 10 μmol/L 5-(and-6)-chloromethyl-2â€²,7â€²-dichorodihydro-fluorescein diacetate-acetoxymethyl ester (DCFDA-AM) for 10 min at 37°C. A 50 μL aliquot was sonicated and used for the determination of the cell proteins. The protein content of cell lysates was assessed using the BCA kit (Sigma-Aldrich). The remaining suspension of cells was washed five times with PBS and re-suspended in 0.5 mL PBS. The intracellular fluorescence of DCFDA was detected (λ excitation = 504 nm, λ emission = 530 nm) using a Synergy HT microplate reader (Bio-Tek Instruments). The fluorescence value was normalized for the protein content and expressed as nmol DCFDA/mg cell proteins, according to the titration curve previously set.

### Metabolite analysis

All metabolite analysis and quantification enzyme activities are described in the Supplementary Materials and Methods.

### The pentose phosphate pathway (PPP)

Cells were washed with fresh medium, detached with trypsin/EDTA (0.05/0.02% v/v), washed with PBS, and resuspended in 1 ml Hepes buffer (145 mmol/L NaCl, 5 mmol/L KCl, 1 mmol/L MgSO_4_, 10 mmol/L Hepes, 10 mmol/L glucose, 1 mmol/L CaCl_2_, pH 7.4) containing 2 μCi of [6-^14^C] glucose (55 mCi/mmol, PerkinElmer) or 2 μCi of [1-^14^C] glucose (58 mCi/mmol, PerkinElmer). A 50 μL aliquot was sonicated and used for the determination of the cell proteins. The remaining suspension of cells was incubated for 1 h in a closed experimental system to trap the ^14^CO_2_ produced from the [^14^C] glucose, and the reaction was stopped by injecting 0.5 mL 0.8 N HClO_4_, as described previously (42). ^14^CO_2_ is released when [1-^14^C] glucose is metabolized, either by the PPP or by the TCA, whereas it is only developed from [6-^14^C] glucose only via the TCA. The amount of glucose transformed into CO_2_ through the PPP was calculated as described [42] and expressed as nmol CO_2_/h/mg cell proteins.

### Tricarboxylic acid cycle (TCA)

Cells were treated as reported for the PPP and radiolabeled with 2 μCi of [1-^14^C] acetylcoenzyme A (46 mCi/mmol, PerkinElmer). A 50 μl aliquot was sonicated and used for determination of cell proteins. The remaining suspension of cells was processed as reported above to trap the ^14^CO_2_ produced from the [^14^C] acetylcoenzyme A. The amount of ^14^CO_2_ was measured by liquid scintillation and expressed as pmol CO_2_/h/mg cell proteins.

### Fatty acid β-oxidation

This assay was performed as previously reported [43]. Cells were washed twice with PBS, detached with trypsin/EDTA (0.05/0.02% v/v) and centrifuged at 13,000 × g for 5 min. A 50 μL aliquot was collected, sonicated and used for intracellular protein quantification. The remaining sample was re-suspended in culture medium containing 0.24 mmol/L fatty acid-free bovine serum albumin, 0.5 mmol/L L-carnitine, 20 mmol/L Hepes, 2 μCi [1-^14^C] palmitic acid (3.3 mCi/mmol, PerkinElmer) and transferred into test tubes that were tightly sealed with rubber caps. In each experimental set, cells were pre-incubated for 30 min with the carnitine palmitoyltransferase inhibitor etomoxir (1 μmol/L) or with the AMP-kinase activator 5-aminoimidazole-4-carboxamide ribonucleotide AICAR (1 mmol/L), as negative and positive controls, respectively. After a 2 h-incubation at 37°C, 0.3 mL of a 1:1 v/v phenylethylamine/methanol solution was added to each sample using a syringe, followed by 0.3 mL 0.8 N HClO_4_. Samples were incubated for a further 1 hr at room temperature, then centrifuged at 13,000 × g for 10 min. The supernatants, containing ^14^CO_2_, and the precipitates, containing ^14^C-acid soluble metabolites (ASM), were collected. The radioactivity of each sample was counted by liquid scintillation. Results were expressed as pmol of [^14^CO_2_] or ^14^C-ASM/h/mg cell proteins.

### Oxygen consumption

Oxygen consumption was measured on 50,000 living cells with the Oxygen Consumption Rate assay Kit (Cayman Chemical, Ann Arbor, MI), following the manufacturer's instructions. Results were expressed as the lifetime signal of the fluorescent probe MitoXpress provided in the kit, versus the assay duration.

### ATP detection

The ATP level in mitochondria extracts, obtained as reported in the Supplementary Materials and Methods, was measured with the ATP Bioluminescent Assay Kit (Sigma-Aldrich), using a Synergy HT Multi-Mode Microplate Reader (Bio-Tek Instruments). ATP was quantified as relative light units (RLU) and converted into nmol ATP/mg mitochondrial proteins, according to the calibration curve previously set.

### Enolase enzymatic activity assay

Quantification of enolase enzymatic activity is described in the Supplementary Materials and Methods.

### Cell cycle analysis

Cells (2 × 10^5^) were cultured for 24 hr in serum-free medium and for a further 24 or 48 hr in complete medium. After ethanol fixation, cells were washed with PBS, suspended in PBS containing 2 μg/ml propidium iodide (Sigma) plus 20 mg/ml RNAse (Life Technologies), and acquired using a FACSCalibur instrument and CellQuest software (BD Biosciences). Cell cycle was analyzed using ModFit software (BD Biosciences).

### Analysis of SA-β-gal activity

SA-β-gal was stained using the Senescence Associated β-Galactosidase Staining kit (Cell Signaling) at pH6, according to the manufacturer's protocol. Images reflect representative results of at least three independent experiments. For antioxidant treatment, PT45 cells were treated daily with NAC or TROLOX-C and SA-β-gal was stained after 7 days.

### *In vivo* studies

All animals were treated in accordance with European and institutional guidelines (Legislative Order No. 116/92). SCID-beige mice (Harlan) of 6 weeks old were allowed to acclimate for 1 week in the animal facility before any intervention was initiated. shCTRL or shENO1 CFPAC-1 and MDA-MB-231 tumor cells (6.5 × 10^5^ and 2 × 10^6^ cells in 100 μL PBS, respectively) were injected subcutaneously into the flank. In order to determine tumor volume by external caliper, the greatest longitudinal diameter (length) and the greatest transverse diameter (width) were measured. Tumor volumes based on caliper measurements were calculated. When tumors reached 1 cm^3^ in volume, animals were sacrificed for ethical reasons.

### Statistical analysis

Statistical analyses were performed (with GraphPad Prism 5) using the unpaired Student's *t* test and two-way ANOVA with Sidak's post hoc test, as appropriate. For all experiments with error bars, SEM was calculated to indicate the variation within each experiment and data, and reported values represent the mean ± SEM.

## SUPPLEMENTARY MATERIALS FIGURES AND TABLES




