# Supplementary material for: Targeting the Warburg effect in cancer cells through ENO1 knockdown rescues oxidative phosphorylation and induces growth arrest
Source: Oncotarget. 2015 Dec 30;7(5):5598–612. doi: 10.18632/oncotarget.6798 (PMC4868708; doi:10.18632/oncotarget.6798)
Supplement: Supplementary file 1 [file oncotarget-07-5598-s001.pdf]

Targeting the Warburg effect in cancer cells through ENO1 knockdown rescues oxidative phosphorylation and induces growth arrest

Supplementary Materials

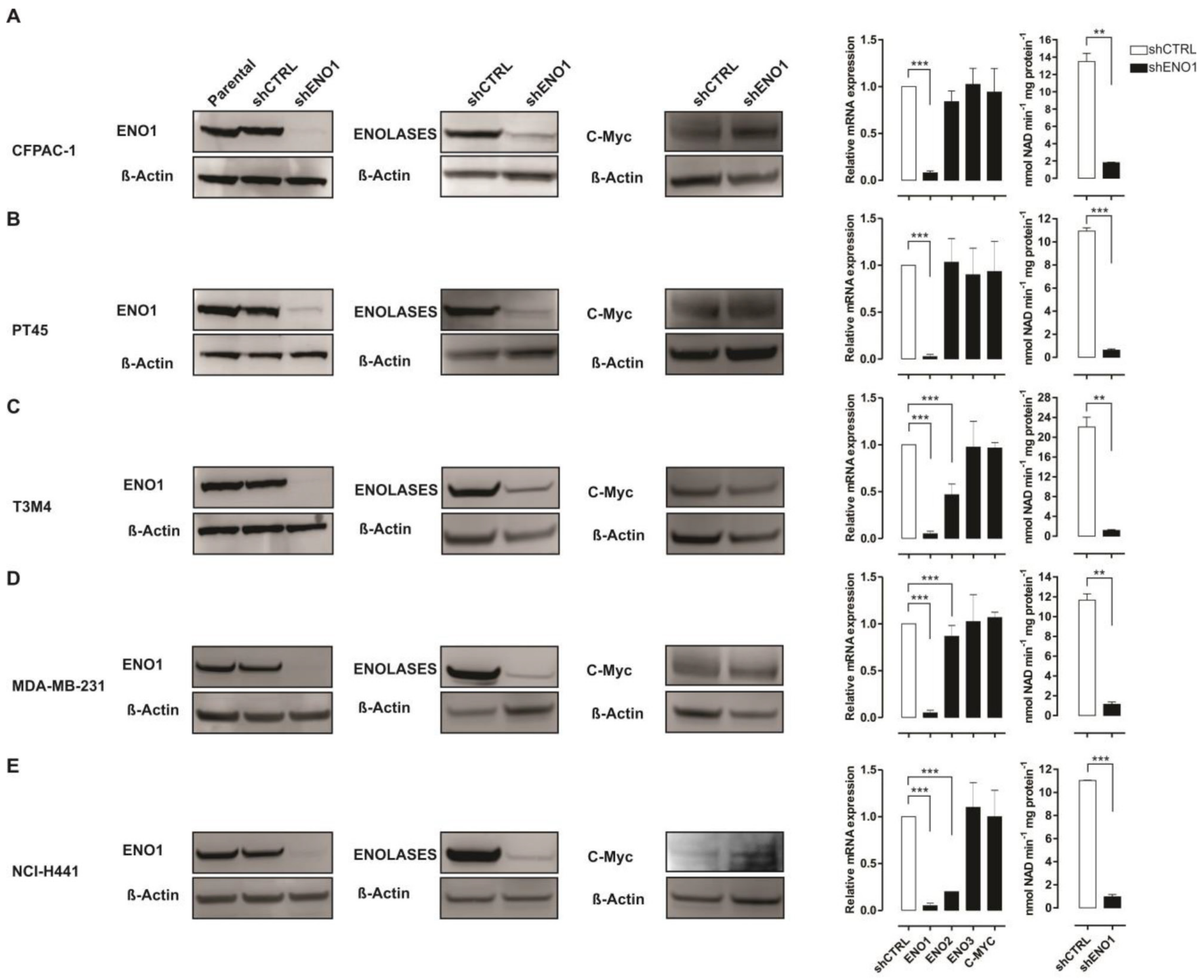

Supplementary Figure S1: ENO1 gene silencing in a panel of human cancer cells. Western blot analysis of ENO1 (left panels), all enolase isoforms (assessed by using a pan-enolase polyclonal antibody, middle left panels) and c-Myc expression (middle panels) in CFPAC-1 (**A**) PT45 (**B**) T3M4 (**C**) MDA-MB-231 (**D**) and NCI-H441 (**E**) non-transduced (parental) and stably transduced with an shRNA targeting ENO1 (shENO1) or a scrambled shRNA (shCTRL) as a control. β-actin was used as loading control. qRT-PCR analysis (middle right panels) of ENO1, ENO2, ENO3 and c-Myc and analysis of enolase activity measured as the rate of NADH oxidation (right panels) in CFPAC-1 (A), PT45 (B), T3M4 (C), MDA-MB-231 (D) and NCI-H441 (E) shCTRL and shENO1 cells. Results are the mean of three independent experiments ± SEM of triplicates. ***$p < 0.001$ relative to shCTRL. ENO1 knockdown also affects ENO2 and ENO3 expression, most probably through post-translational mechanisms, as ENO2 and ENO3 mRNA was unaffected. The expression of c-Myc, which is transcriptionally repressed by the ENO1 alternative transcript MBP-1, was not influenced by ENO1 knockdown, ruling out any functional effects related to this gene in ENO1-silenced cells.

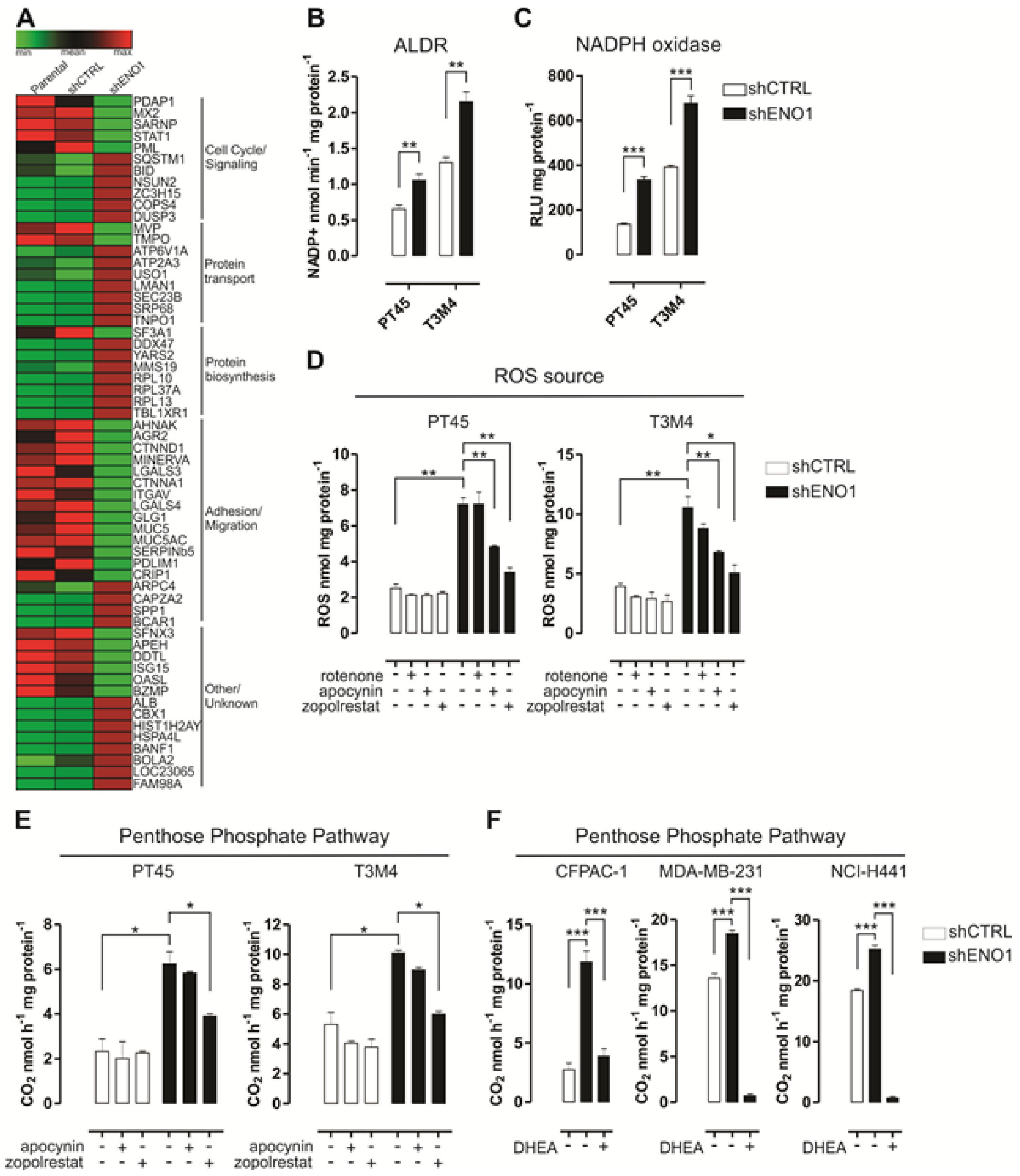

Supplementary Figure S2: The polyol and pentose phosphate pathways increase the concentration of intracellular reactive oxygen species (ROS) in ENO1-silenced cells. (**A**) Heat map of differentially-expressed proteins in shENO1 compared to shCTRL CFPAC-1 cells. Based on the spectra count label-free quantitation approach, LC-MS/MS analysis identified 32 up-regulated (red) and 28 down-regulated (green) proteins. Proteins are classified according to their function: cell cycle/signaling, protein transport, protein biosynthesis, cell adhesion/migration and others/unknown. See also Figure 1A for other identified proteins. (**B–C**) Analysis of aldose reductase (ALDR) activity measured as the rate of NADPH oxidation (B), and NADPH oxidase activity assessed by the isoluminol-chemiluminescence assay (C) in shCTRL and shENO1 PT45 and T3M4 cell lines. Chemiluminescence was expressed as relative luminescence unit (RLU)/mg cell proteins. (**D–E**) Analysis of ROS concentration measured by DCFDA-AM assay (D) and of [1-^{14}C] glucose flux through the Pentose Phosphate Pathway (PPP) assessed through $^{14}CO_2$ release (E) after selective inhibition of mitochondrial chain (rotenone), NADPH oxidase (apocynin) and ALDR (zopolrestat) in shCTRL and shENO1 PT45 (left panels) and T3M4 (right panels) cell lines. (**F**) Analysis of PPP activity, as described above, after selective inhibition of the PPP (DHEA) in CFPAC-1 (left panels), MDA-MB-231 (middle panel) and NCI-H441 (right panel) cell lines. All the graphs illustrate the mean result of three independent experiments ± SEM *$p < 0.05$; **$p < 0.01$;***$p < 0.001$ relative to shCTRL.

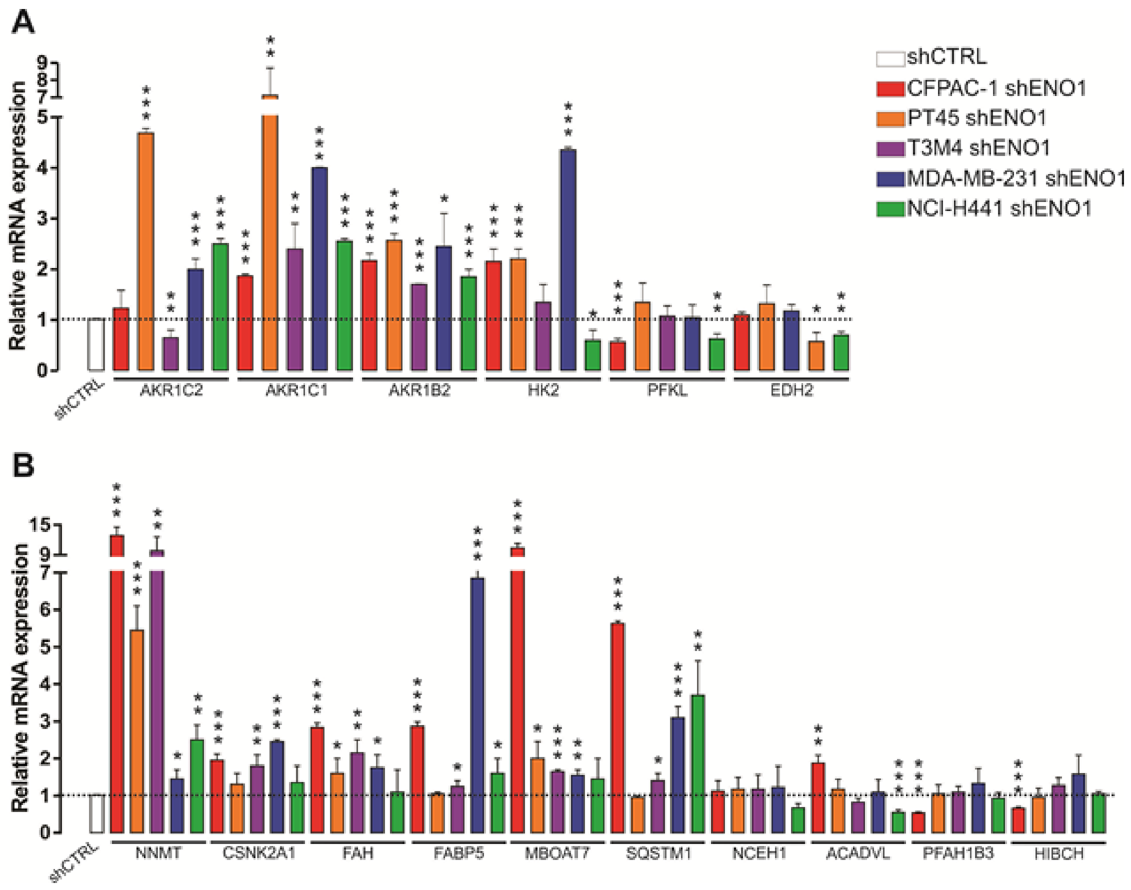

Supplementary Figure S3: mRNA expression analysis of mass spectrometry identified proteins. (**A–B**) Transcript analysis of proteins identified as up-regulated or down-regulated through semi-quantitative tandem mass spectrometry analysis in CFPAC-1 (red), PT45 (orange), T3M4 (purple), MDA-MB-231 (blue) and NCI-H441 (green) shENO1 compared to shCTRL (white) cells. Genes were divided according to their function: glycolysis-related (A) and catabolic pathways (B). Results are the mean ± SEM of triplicates. $*p < 0.05$; $**p < 0.01$; $***p < 0.001$ relative to shCTRL-transduced cells. See Table S2 and S3 for extended gene names.

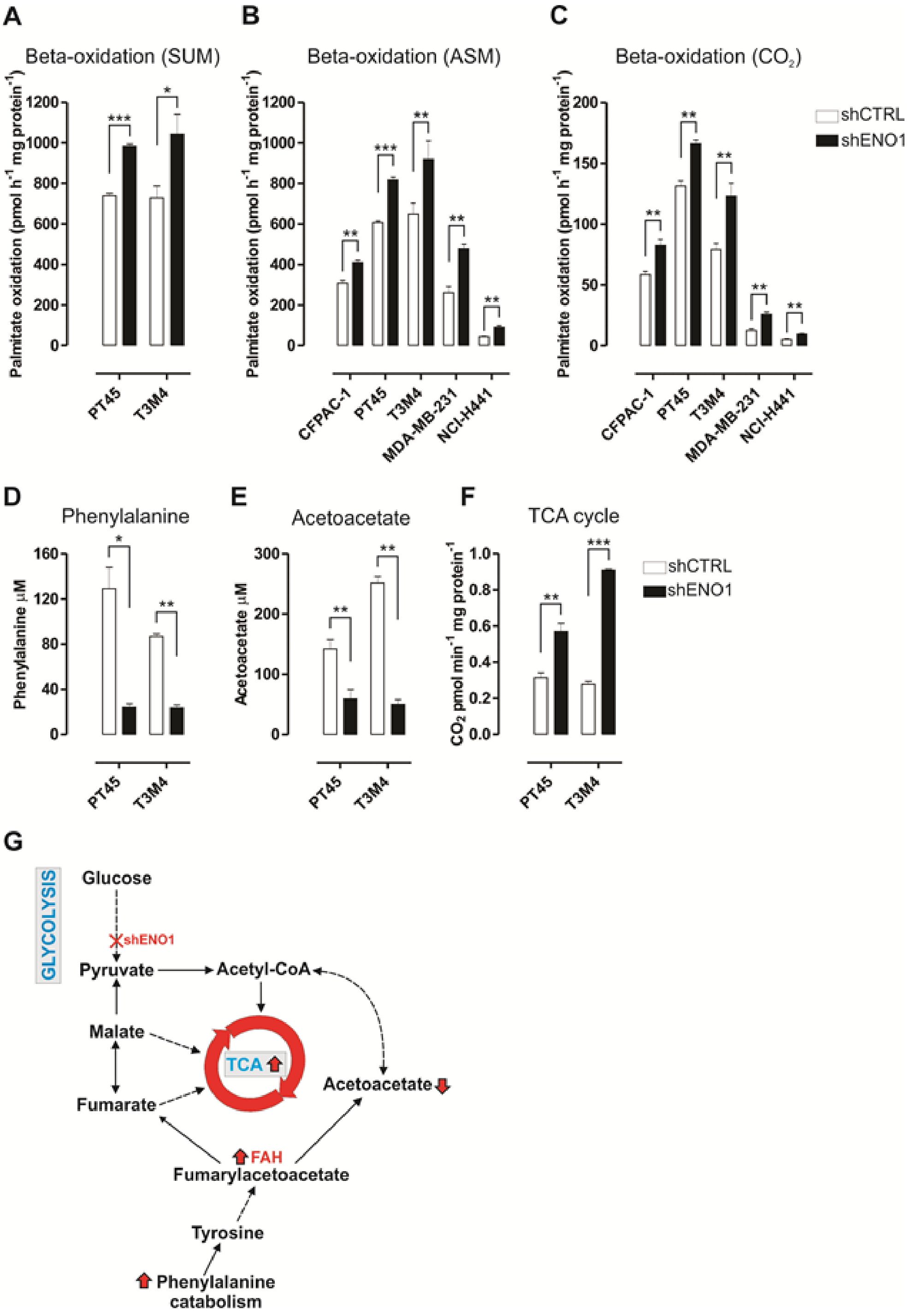

Supplementary Figure S4: ENO1 silencing enhances catabolic pathway adaptations. (**A–C**) Cells were exposed to [1-^{14}C] palmitic acid and total palmitate oxidation (sum of ^{14}C-acid soluble metabolites and $^{14}CO_2$ production) of palmitic acid in shCTRL and shENO1 PT45 and T3M4 cell lines (A) was measured. ^{14}C-acid soluble metabolites production (B) and $^{14}CO_2$ production (C) were measured in shCTRL and shENO1 CFPAC-1, PT45, T3M4, MDA-MB-231 and NCI-H441 cell lines. (**D–E**) Analysis of phenylalanine (D) and acetoacetate (E) concentration in shCTRL and shENO1 PT45 and T3M4 cell lines. (**F**) The TCA cycle rate was evaluated measuring CO_2 emission after radiolabelling cells with [1-^{14}C] acetylcoenzyme A in shCTRL and shENO1 PT45 and T3M4 cell lines. All the graphs illustrate the mean result of three independent experiments ± SEM *$p < 0.05$; **$p < 0.01$;***$p < 0.001$ relative to shCTRL. (**G**) Schematic diagram of ENO1 silencing-induced catabolic pathway adaptations.

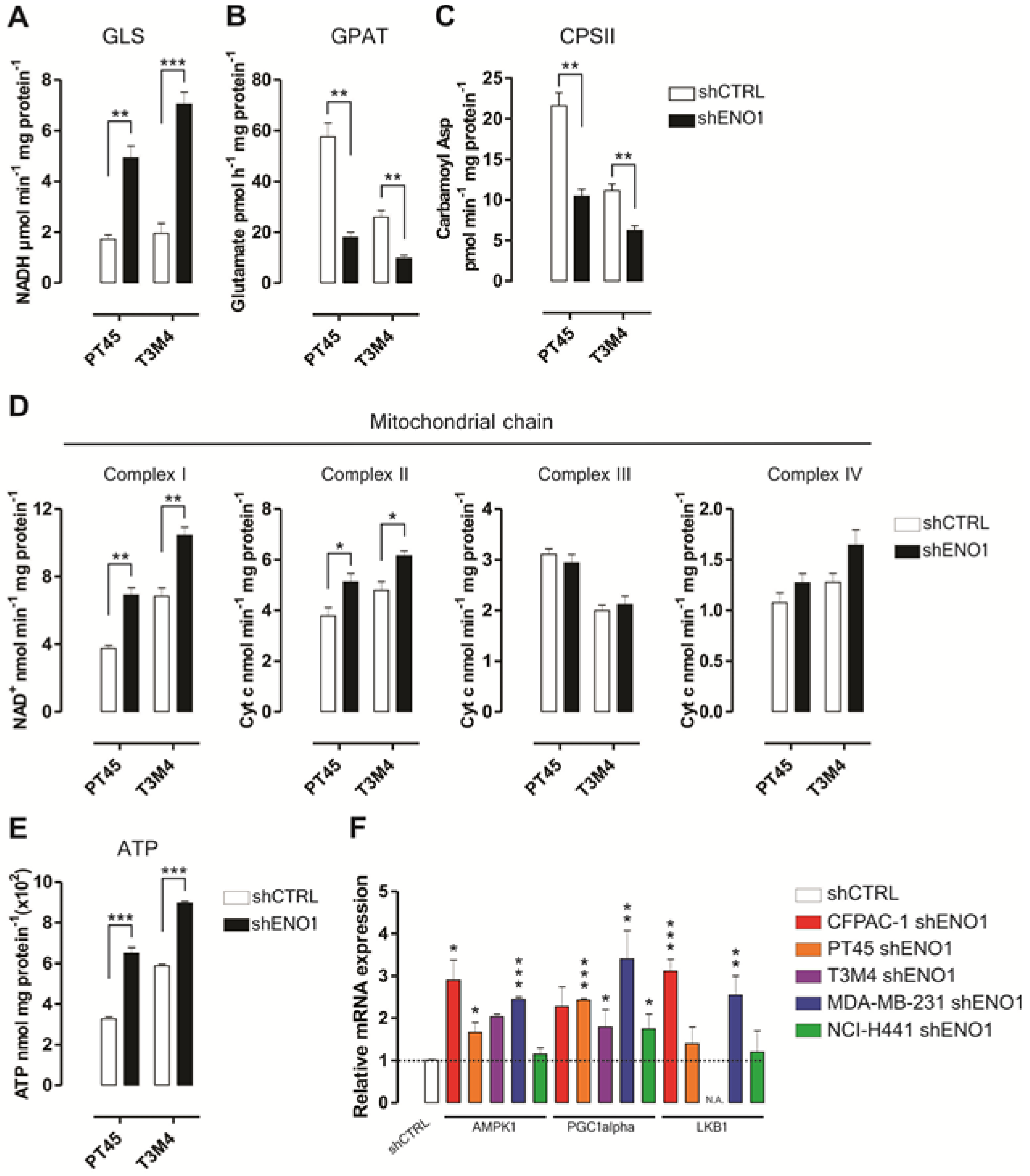

Supplementary Figure S5: ENO1 knockdown promotes oxidative phosphorylation. (**A–C**) Analysis of glutaminase (GLS) (A), glutamine amidophosphoribosyltransferase (GPAT) (B) and carbamoyl phosphate synthetase II (CPSII) (C) activity in shCTRL and shENO1 PT45 and T3M4 cell lines. (**D**) Analysis of mitochondrial respiratory chain complex I and complexes II–IV activity in shCTRL and shENO1 PT45 and T3M4 cells, expressed as nmol NAD^+/min/mg mitochondrial protein for complex I, nmol Cyt c reduced/min/mg mitochondrial proteins for complexes II–III and nmol Cyt c oxidized/min/mg mitochondrial protein for complex IV. (**E**) Analysis of ATP production in shCTRL and shENO1 PT45 and T3M4 cell lines. All the graphs illustrate the mean result of three independent experiments ± SEM. $*p < 0.05$; $**p < 0.01$;$***p < 0.001$ relative to shCTRL. (**F**) Transcript analysis of proteins involved in catabolic pathways/ mitochondrial biogenesis in CFPAC-1 (red), PT45 (orange), T3M4 (purple), MDA-MB-231 (blue) and NCI-H441 (green) shENO1 compared to shCTRL (white) cells. Results are the mean ± SEM of triplicates. $*p < 0.05$; $**p < 0.01$;$***p < 0.001$ relative to shCTRL-transduced cells.

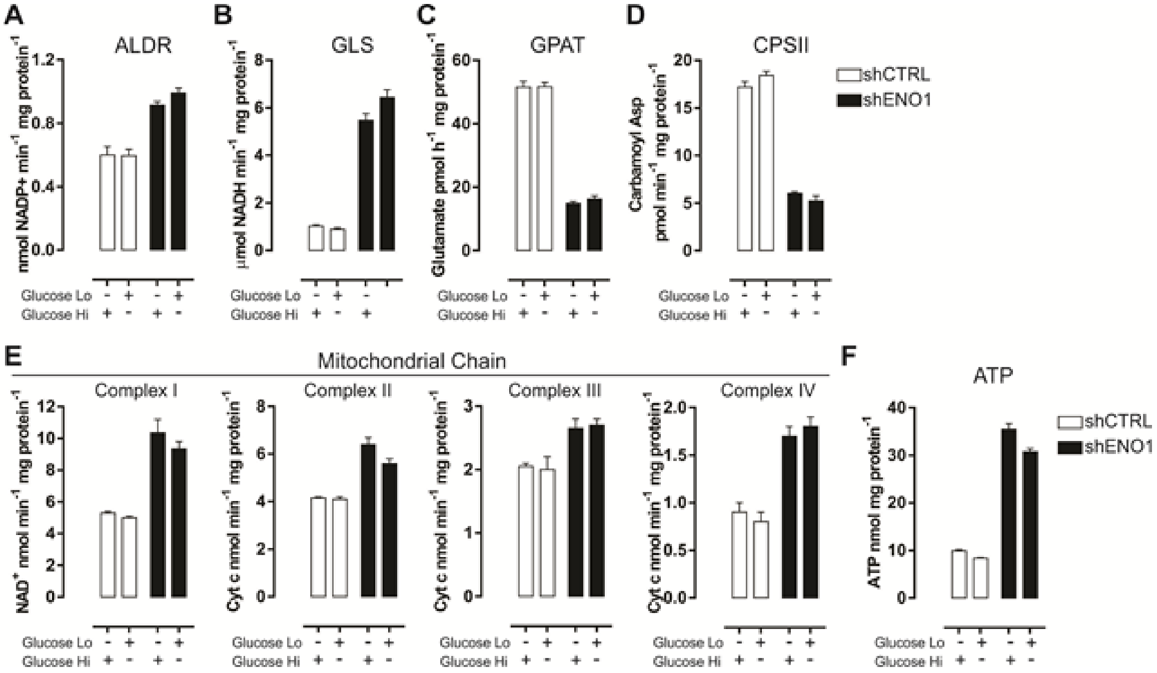

Supplementary Figure S6: Analysis of ENO1-silenced cell metabolic adaptations under low glucose conditions. (**A–F**) Analysis of aldose reductase (ALDR) (A), glutaminase (GLS) (B), glutamine amidophosphoribosyltransferase (GPAT) (C), carbamoyl phosphate synthetase II (CPSII) (D), mitochondrial respiratory chain complex I and complexes II-IV (E) activity and ATP production (F) in shCTRL (white bars) or shENO1 (black bars) CFPAC-1 cells after 24 h culture in low (1 g/L) or high (4.5 g/L) glucose media. Results are mean of three independent experiments ± SEM.

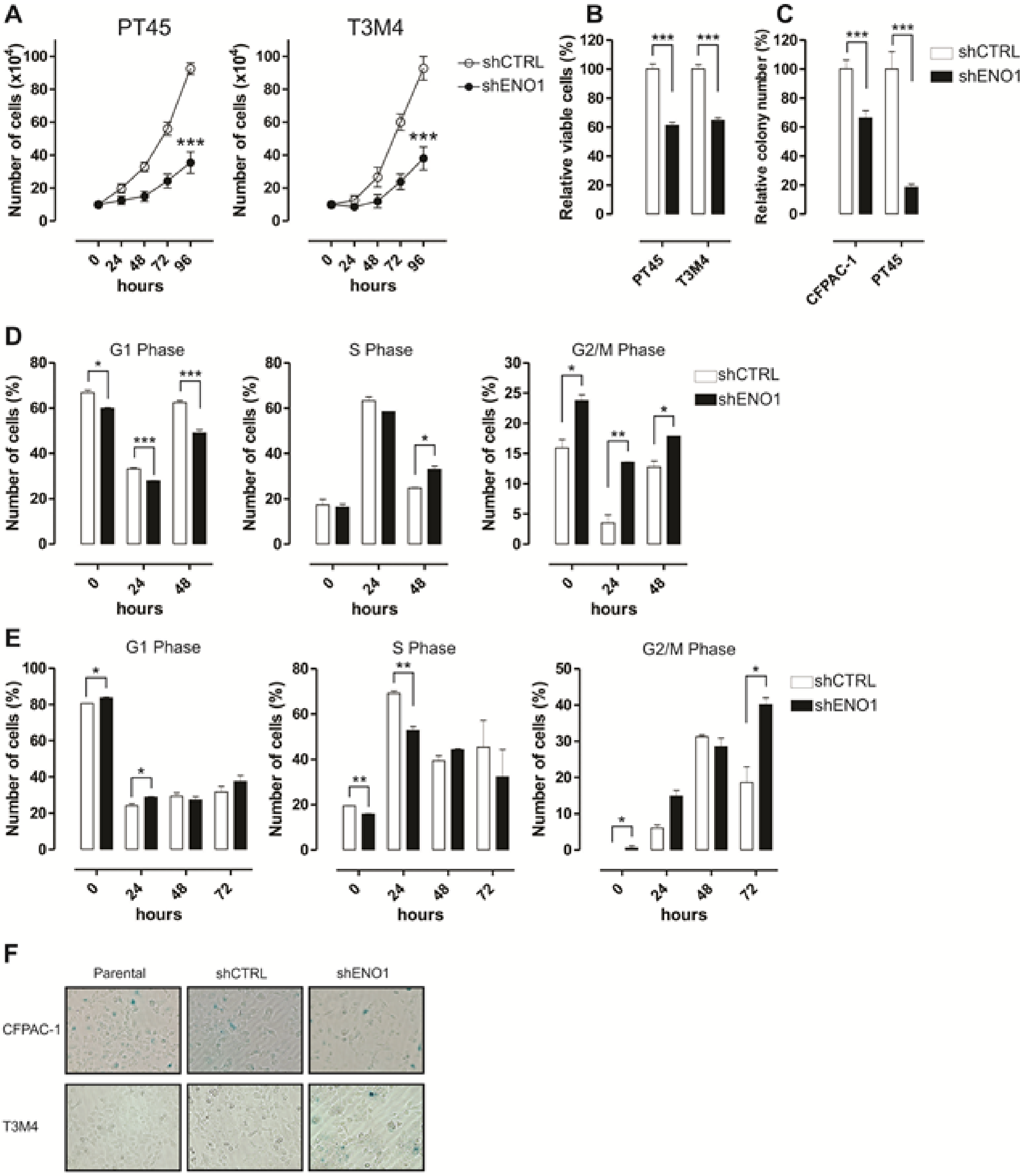

Supplementary Figure S7: ENO1 silencing impairs cancer cell growth. (**A**) Cell proliferation analysis of PT45 (left panel) and T3M4 (right panel) after shCTRL or shENO1 infection. Cells were starved and counted every 24 hr after serum replenishment. ***p < 0.001 was assessed by two-way ANOVA with Sidak's post hoc test. (**B**) Cell survival assessed by MTT assay. Cells were starved and MTT solution was added 48 hr after serum replenishment. OD values were measured at 570 nm. (**C**) Colony forming assay in soft agar. Cells were plated in 0.45% agarose overlaying a 0.9% agar layer. Colonies were counted by optical microscope after 3 weeks. T3M4 cell line was unable to form colonies in soft agar. (**D–E**) Flow cytometry cell cycle analysis of serum-starved shCTRL and shENO1 NCI-H441 (D) and MDA-MB-231 (E) cells at the indicated time points after serum replenishment. Data are expressed as the percentage of cells at each phase. (**F**) Senescence-associated β-galactosidase staining. Senescent CFPAC-1 and T3M4 cells were colored blue upon X-gal staining at pH 6. Parental and shCTRL CFPAC-1 cells showed positive β-galactosidase staining even in the absence of a senescent morphology. One representative out of three independent experiments is shown. Results are mean of three independent experiments ± SEM. *p < 0.05, **p < 0.01, ***p < 0.001 relative to shCTRL.

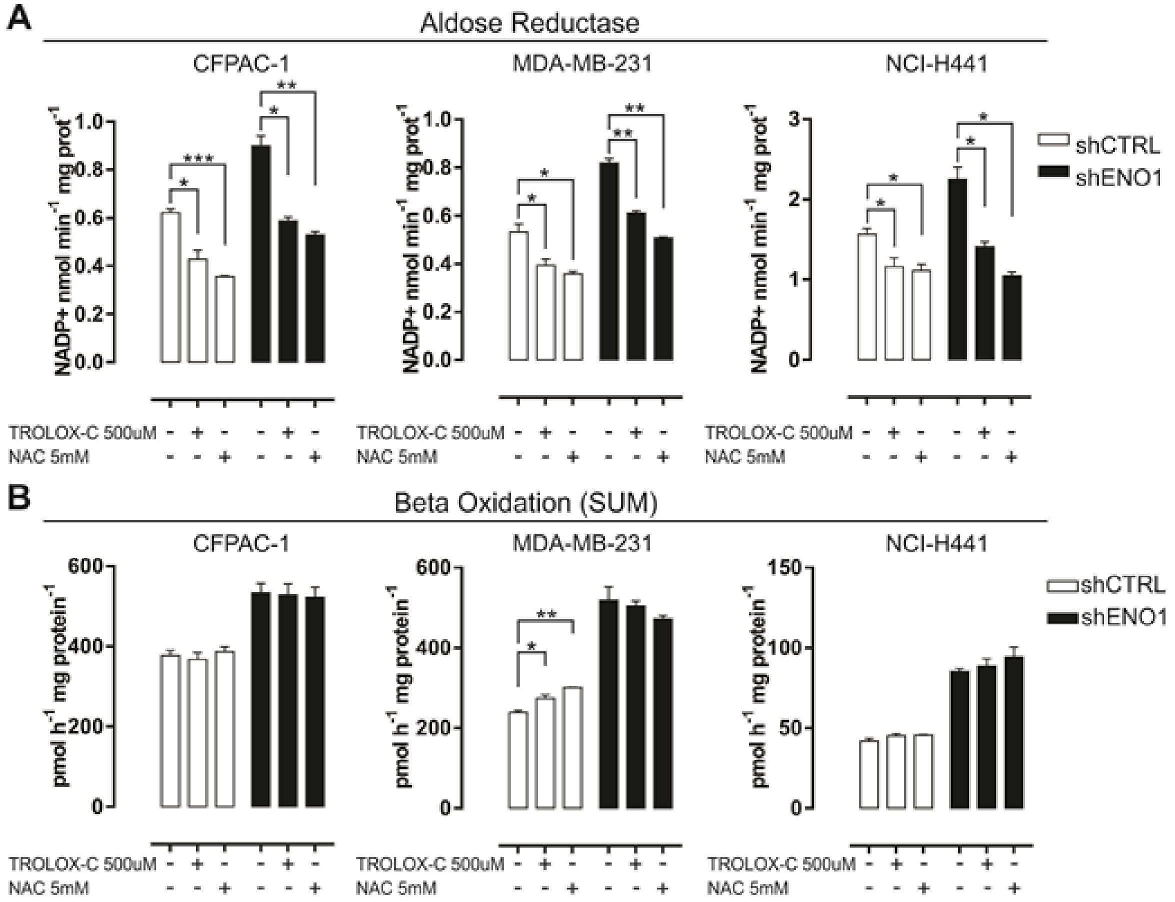

Supplementary Figure S8: Analysis of ENO1-silenced cell metabolic adaptations under antioxidant treatment. (**A**) Analysis of aldose reductase (ALDR) activity measured through the rate of NADPH oxidation in shCTRL (white bars) and shENO1 (black bars) cell lines after 24 h treatment with NAC or TROLOX-C. (**B**) Oxidation of palmitic acid in CFPAC-1, MDA-MB-231 and NCI-H441 cell lines transduced with shCTRL (white bars) or shENO1 (black bars) after 24 h treatment with antioxidants. Results are mean of three independent experiments ± SEM. $^*p < 0.05$, $^{**}p < 0.01$, $^{***}p < 0.001$ relative to untreated cells.

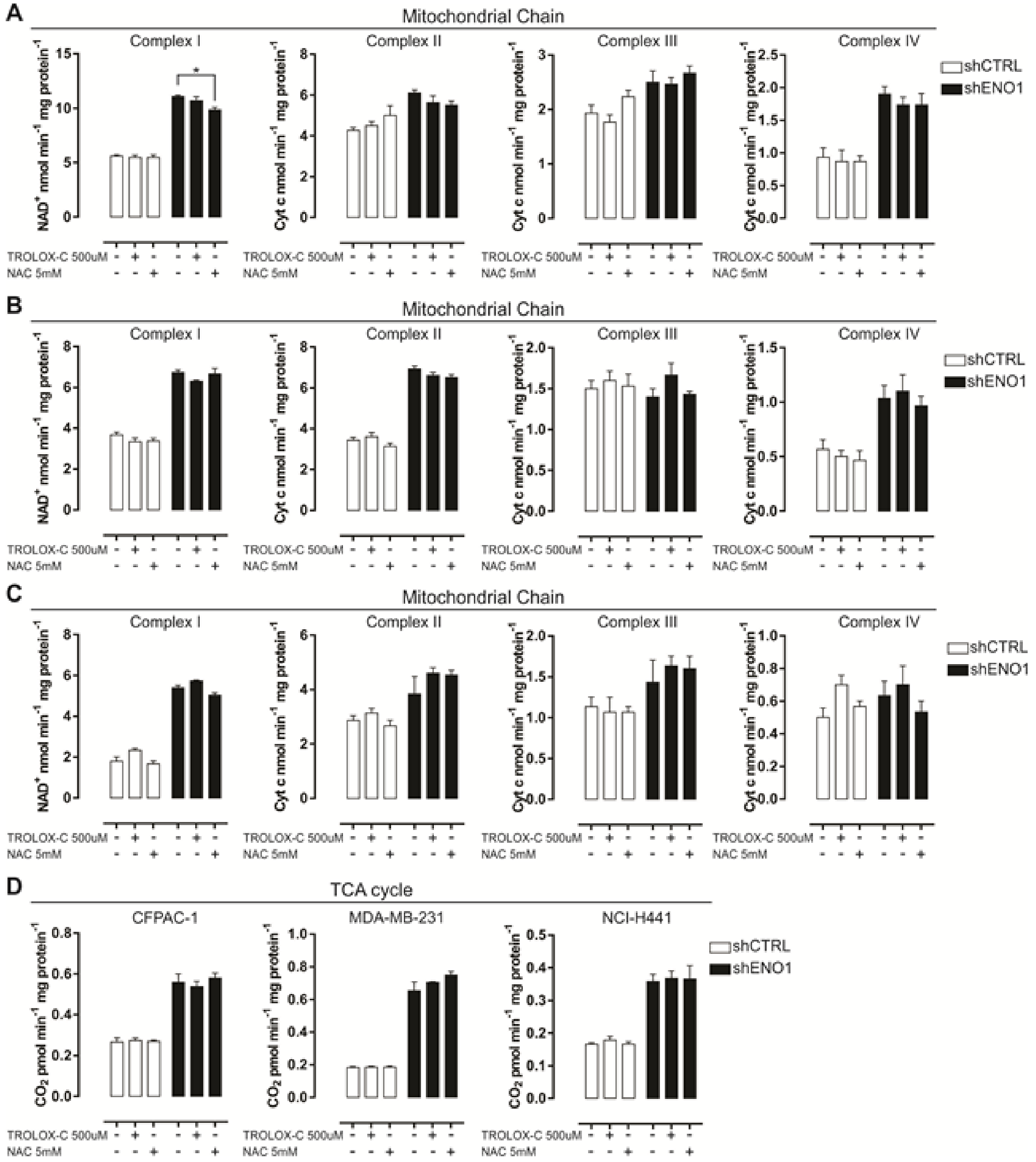

Supplementary Figure S9: Analysis of ENO1-silenced cell metabolic adaptations under antioxidant treatment. (**A–C**) Analysis of mitochondrial respiratory chain complex I and complexes II–IV activity in CFPAC-1 (A), MDA-MB-231 (B) and NCI-H441 (C) cell lines after after 24 h treatment with NAC or TROLOX-C. Activity is expressed as nmol NAD^+/min/mg mitochondrial protein for complex I; nmol Cyt c reduced/min/mg mitochondrial protein for complexes II–III and nmol Cyt c oxidized/min/mg mitochondrial protein for complex IV. (**D**) The TCA cycle rate was evaluated measuring CO_2 emission after radiolabelling cells with [1-^{14}C] acetylcoenzyme A in CFPAC-1, MDA-MB-231 and NCI-H441 cell lines treated for 24 h with antioxidants. TCA cycle activity is expressed as pmol CO_2/min/mg protein. Results are mean of three independent experiments ± SEM.

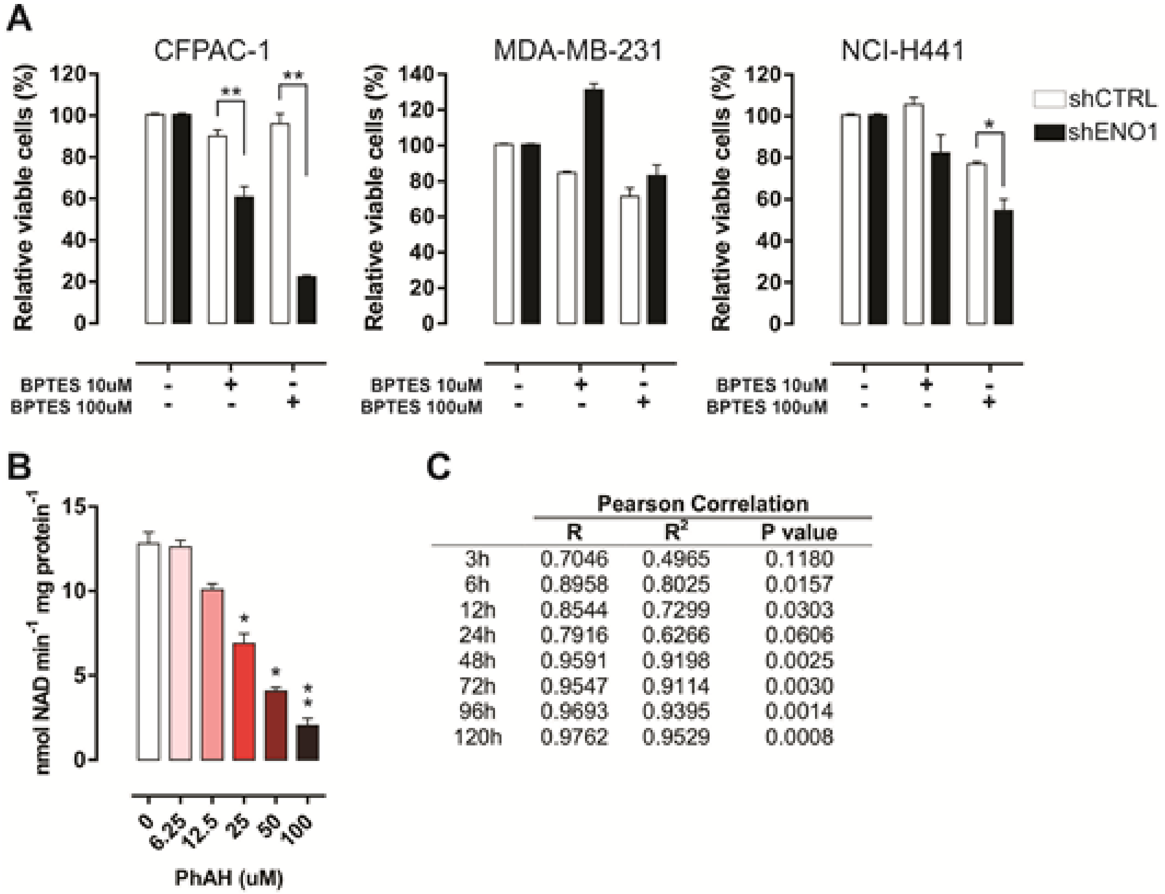

	Pearson Correlation		
	R	R^2	P value
3h	0.7046	0.4965	0.1180
6h	0.8958	0.8025	0.0157
12h	0.8544	0.7299	0.0303
24h	0.7916	0.6266	0.0606
48h	0.9591	0.9198	0.0025
72h	0.9547	0.9114	0.0030
96h	0.9693	0.9395	0.0014
120h	0.9762	0.9529	0.0008

Supplementary Figure S10: (**A**) Effects of GLS inhibitor BPTES (bis-2-(5phenylacetamido-1,2,4-thiadiazol-2-yl) ethyl sulfide) on shENO1 cells after 96 h treatment as assessed by MTT assay. (**B**) Dose-dependent analysis of ENO1 enzymatic activity in CFPAC-1 cells after 6 h treatment with PhAH. Results are mean of three independent experiments ± SEM. *$p < 0.05$, **$p < 0.01$, ***$p < 0.001$ relative to untreated. (**C**) Pearson's correlation analysis between CFPAC-1 cell enolase enzymatic activity shown in panel B and the percentage of viable cells compared to the untreated control after treatment with different doses of PhAH (range 6.25 – 100 μM, shown in Figure 6G) for the indicated time.

Supplementary Table S1: Complete MS analysis and criteria (Excel file)

Supplementary Table S2: Upregulated proteins

Protein name	Functional annotation	Accession[a]	Average Spectra Count (MS2#) Parental	shCTRL	shENO1	Relative Difference (%)[b]
aldo-keto reductase family 1, member C2 (AKR1C2)	metabolism	4503285	14	6	29	131
aldo-keto reductase family 1, member C1 (AKR1C1)	metabolism	5453543	2	2	11	138
nicotinamide N-methyltransferase (NNMT)	metabolism	5453790	2	2	6	100
casein kinase II alpha 1 subunit isoform a (CSNK2A1)	metabolism/cell cycle	29570791	1	1	5	133
aldo-keto reductase family 1, member B1 (AKR1B1)	metabolism	4502049	0	0	5	200
inorganic pyrophosphatase 2 isoform 1 precursor (PPA2)	metabolism	29171702	1	1	4	120
ilvB (bacterial acetolactate synthase)-like (ILVBL)	metabolism	21361361	0	0	3	200
carbonyl reductase 1 (CBR1)	metabolism/ antioxidant	4502599	4	4	12	100
nitric oxide synthase interacting protein (NOSIP)	NO metabolism	7705716	0	0	3	200
hexokinase 2 (HK2)	glycolysis	15553127	0	0	5	200
fumarylacetoacetase (FAH)	tyrosine catabolism	4557587	0	0	3	200
albumin preproprotein (ALB)	starvation response	4502027	0	0	6	200
fatty acid binding protein 5 (psoriasis-associated) (FABP5)	lipid transport	4557581	5	3	16	137
leukocyte receptor cluster member 4 protein (MBOAT7)	lipid metabolism	23308572	0	1	6	143
sequestosome 1 isoform 1 (SQSTM1)	autophagy/apoptosis	4505571	6	2	20	164
BH3 interacting domain death agonist isoform 2 (BID)	apoptosis	4557361	1	0	4	200
DEAD (Asp-Glu-Ala-Asp) box polypeptide 47 isoform 1 (DDX47)	apoptosis/mRNA processing	20149629	0	0	3	200
NOL1/NOP2/Sun domain family, member 2 (NSUN2)	cell cycle	39995082	1	1	6	143
erythropoietin 4 immediate early response (ZC3H15)	cell cycle	118150660	1	1	4	120
COP9 signalosome subunit 4 (COPS4)	cell cycle	38373690	2	2	6	100
dual specificity phosphatase 3 (DUSP3)	signaling	4758208	1	1	6	143
ATPase, H+ transporting, lysosomal V1 subunit A (ATP6V1A)	vacuolar H^+ ion transport	19913424	3	4	17	124
ATPase, Ca^{++} transporting, slow twitch 2 isoform 3 (ATP2A3)	Ca^{++} ion transporter	209413709	1	0	6	200
USO1 homolog, vesicle docking protein (USO1)	vescicular transport	4505541	1	0	5	200

lectin, mannose-binding, 1 precursor (LMAN1)	protein transport	5031873	0	0	3	200
Sec23 homolog B (SEC23B)	protein transport	14591928	0	0	3	200
signal recognition particle 68kDa (SRP68)	protein transport	24497620	0	0	3	200
transportin 1 isoform 1 (TNPO1)	protein transport	133925811	0	0	3	200
actin related protein 2/3 complex subunit 4 isoform a (ARPC4)	cytoskeleton organization	5031595	1	0	4	200
capping protein (actin filament) muscle Z-line, alpha 2 (CAPZA2)	cytoskeleton organization	5453599	0	0	3	200
secreted phosphoprotein 1 isoform a (SPP1)	cell adhesion	91206462	0	0	4	200
breast cancer anti-estrogen resistance 1 (BCAR1)	cell adhesion	44662836	0	0	3	200
tyrosyl-tRNA synthetase 2, mitochondrial (YARS2)	protein synthesis	94681057	0	0	5	200
MMS19 nucleotide excision repair homolog (MMS19)	transcription/DNA damage	170763479	2	1	10	164
ribosomal protein L10 (RPL10)	translation	223890243	0	0	4	200
ribosomal protein L37a (RPL37A)	translation	4506643	0	0	3	200
ribosomal protein L13a (RPL13)	translation	6912634	0	0	3	200
transducin (beta)-like 1 X-linked receptor 1 (TBL1XR1)	transcription	19913371	0	0	4	200
heterochromatin protein 1-beta (CBX1)	heterocromatin	187960037	0	0	4	200
H2A histone family, member Y isoform 1 (HIST1H2AY)	nucleosome assembly	20336746	0	0	3	200
heat shock 70kDa protein 4-like (HSPA4L)	stress response	31541941	0	0	4	200
barrier to autointegration factor 1 (BANF1)	antiviral response	4502389	0	0	3	200
bolA-like protein 2B (BOLA2)	ND	85797673	0	1	4	120
hypothetical protein LOC23065	ND	22095331	0	0	4	200
hypothetical protein LOC25940 (FAM98A)	ND	56699482	0	0	4	200

[a]NCBI GI Number.

[b]Relative difference (%) = (MS2# shENO1-MS2#_shCTRL)/[(MS2#_shENO1+ MS2#_shCTRL)/2]*100.

Supplementary Table S3: Downregulated proteins

Protein name	Functional annotation	Accession[a]	Average Spectra Count (MS2#) Parental	shCTRL	shENO1	Relative Difference (%)[b]
enolase 1 (ENO1)	glycolysis	4503571	124	117	23	–134
enolase 2 (ENO2)	glycolysis	5803011	145	151	8	–180
liver phosphofructokinase (PFKL)	glycolysis	48762920	3	5	0	–200
thiosulfate sulfurtransferase (TST)	metabolism	17402865	4	8	0	–200
EH-domain containing 2 (EHD2)	endocytosis/ metabolism	21361462	6	7	2	–111
arylacetamide deacetylase-like 1 isoform b (NCEH1)	lipid metabolism	68051721	6	7	2	–111
acyl-Coenzyme A dehydrogenase, very long chain isoform 2 precursor (ACADVL)	lipid metabolism	76496475	4	6	0	–200
acyl-CoA thioesterase 7 isoform hBACHa (ACOT7)	lipid metabolism	75709208	4	4	0	–200
platelet-activating factor acetylhydrolase, isoform Ib, gamma subunit (PAFAH1B3)	lipid metabolism	4505587	3	4	0	–200
sideroflexin 3 (SFXN3)	iron homeostasis	31621303	4	5	0	–200
3-hydroxyisobutyryl-Coenzyme A hydrolase isoform 1 (HIBCH)	Amino acid catabolism	37594471	4	4	0	–200
AHNAK nucleoprotein isoform 1 (AHNAK)	cell adhesion	61743954	95	110	35	–103
anterior gradient 2 homolog (AGR2)	cell adhesion/ proliferation	5453541	55	82	22	–115
catenin, delta 1 isoform 1ABC (CTNND1)	cell adhesion/ signaling	146231940	8	10	2	–133
hypothetical protein LOC64855 isoform 2 (MINERVA)	cell adhesion	79750824	8	10	1	–164
galectin 3 (LGALS3)	cell adhesion	115430223	13	9	1	–160
catenin, alpha 1 (CTNNA1)	cell adhesion	55770844	6	7	1	–150
integrin alpha-V isoform 1 precursor (ITGAV)	cell adhesion	4504763	5	4	1	–120
galectin-4 (LGALS4)	cell adhesion	5453712	10	13	0	–200
Golgi apparatus protein 1 isoform 1 (GLG1)	cell adhesion/ signaling	54633312	8	13	0	–200
PREDICTED: similar to mucin 5, partial	cell adhesion	169202626	7	10	0	–200
mucin 5AC (MUC5AC)	cell adhesion	161019170	5	6	0	–200
serine (or cysteine) proteinase inhibitor, clade B (ovalbumin), member 5 (SERPINb5)	cell migration	167860126	5	4	1	–120
PDZ and LIM domain 1 (PDLIM1)	cytoskeleton regulation	13994151	9	16	2	–156
cysteine-rich protein 1 (intestinal) (CRIP1)	cytoskeleton regulation	4503047	5	3	0	–200
PDGFA associated protein 1 (PDAP1)	cell proliferation	7657441	5	3	0	–200

myxovirus resistance protein 2 (MX2)	cell cycle/protein transport	11342664	7	8	2	–120
cytokine induced protein 29 kDa (SARNP)	cell cycle	32129199	4	4	1	–120
signal transducer and activator of transcription 1 isoform beta (STAT1)	transcription regulation	21536301	21	20	10	–67
promyelocytic leukemia protein isoform 5 (PML)	transcription regulation	109637788	4	8	0	–200
major vault protein (MVP)	mRNA/protein transport	19913412	23	27	11	–84
splicing factor 3a, subunit 1, 120kDa isoform 1 (SF3A1)	mRNA processing	5032087	3	5	0	–200
thymopoietin isoform beta (TMPO)	nuclear envelope organization	73760405	4	4	0	–200
N-acylaminoacyl-peptide hydrolase (APEH)	proteolysis	23510451	4	4	0	–200
D-dopachrome tautomerase-like (DDTL)	lyase	145386559	4	4	0	–200
ISG15 ubiquitin-like modifier (ISG15)	antiviral response	4826774	4	4	0	–200
2′–5′ oligoadenylate synthetase 3 (OASL)	antiviral response	45007007	4	3	0	–200
beta-2-microglobulin precursor (BZMP)	antigen presentation	4757826	4	3	0	–200

[a]NCBI GI Number.
[b]Relative difference (%) = (MS2# shENO1-MS2#_shCTRL)/[(MS2#_shENO1+ MS2#_shCTRL)/2]*100.

MATERIAL AND METHODS

Viral Transduction

Lentiviral infections were performed using HEK293T cells as producers of viral supernatants. HEK-293T cells were grown on 10-cm plates to 70% confluence and co-transfected with shRNA lentiviral DNA and the helper vectors pCMVΔ8.74 and pVSV-G (Clonetech) using the calcium phosphate precipitation method. The medium was harvested 24 hr post-transfection and filtered through a 0.45 μm filter. Supernatants from HEK-293T cultures were used to cross-transduce cancer cells in the presence of 8 μg/mL Polybrene (Sigma-Aldrich), and subsequent clones were selected by puromycin (2 μg/mL).

shRNAs

Short-hairpin RNAs (shRNAs) targeting the human ENO1 mRNA and cloned into the pLKO.1-puro vector were obtained from the human library MISSION® TRC-Hs 1.0. Five shRNAs targeting ENO1 were purchased as glycerol stocks from Sigma-Aldrich (SHCLNG-NM_001428). The efficiency of each shRNA in decreasing the ENO1 mRNA level was assessed by real-time qRT-PCR in transiently transfected cells. The best shRNA (TRCN0000029324) with the targeted sequence 5′-CCGGCGTACCGCTTCCTTAGAACTTCTCGAGA-AGTTCTAAGGAAGCGGTACGTTTTT-3′ was chosen for the generation of stable cell lines. The control shRNA (SHC002, Sigma-Aldrich) generates a scrambled shRNA sequence that does not target any known human gene.

Quantitative RT–PCR

Total RNA was extracted using the RNeasy Mini kit (Qiagen) and reverse transcription was performed from 2 μg of total RNA using iScript cDNA synthesis kit (BioRad) according to the manufacturer's instructions. Quantitative RT–PCR was performed using SYBR Green dye (Life Technologies) on a Thermal iCycler (BioRad). PCR reactions were performed in triplicate and the relative amount of cDNA was calculated by the comparative C_T method using β-actin RNA sequences as a control. Data are represented as mean ± SEM of three or more independent experiments.

Oligonucleotide primer sequences for SybrGreen qRT-PCR

Gene	Sense	Antisense
ENO1	GCCTCCTGCTCAAAGTCAAC	AACGATGAGACACCATGACG
ENO2	AGTGGACCACATCAACTCCACCAT	ACTCCAGCATCAGGTTGTCCAGTT
ENO3	CTCCCATGCTGGAAACAA	TGGCATCCTTCCCATACT
C-MYC	CTCCACTCGGAAGGACTAT	TTGTGTGTTCGCCTCTTG
AKR1C2	GCGTTGCCAGCTCATTGCTCTTAT	AATCCCAGGACAGGCATGAAGTGA
AKR1C1	GCTTTAGAGGCCACCAAATTGGCA	ACTGCCATCTGCAATCTTGCTTCG
AKR1B2	ACACCAGAACGCATTGCTGAGAAC	AGGTACAGCTCAACAAGGCACAGA
HK2	TGCAGCGCATCAAGGAGAACAAAG	ACGGTCTTATGTAGACGCTTGGCA
PFKL	AGTATACGTGGTGCACGATGGCTT	TGCGGATGTTCTCCACAATGGACT
EDH2	CCGGTGGAAGCATTTAGAG	GAATGACGGATGGATGGATG
NNMT	ATGCGCTCAAGAGCAGCTACTACA	CGTTGTTGGCCATGGTGGAAGAAT
CSNK2A1	AGGCAGGAAGAAAGGAAGGAAGGA	AGACACACTTCCACAAGAGCCACT
FAH	TCGGAAGTGTGCATTCATCTCCCA	TCAACGCATTCTCCTTGTCCCTGA
FABP5	ACAGTTCAGCAGCTGCAAGGAAGA	ATACAATCTGGCTTGGCCATTGCG
MBOAT7	TTGGGCAAATTCCCTTTCT	CTTCCTTCCAAGTCTGCAATA
SQSTM1	AAATGGGTCCACCAGGAAACTGGA	TCAACTTCAATGCCCAGAGGGCTA
NCEH1	CGCTATGTCATGGTGAAGTAT	CAAGAGGGATGTCCAGTTTAG
ACADVL	AGAGGCATCATTGCTAAGGCGGTA	TCTCCTGGATCAGCCCAAAGTTGT
PFAH1B3	CGTGGCTGACAGCAAAGATAAGGA	CCGTCACCACCAATGCCAAAGTTA
HIBCH	ATAAAGGGAGCAGGAGGAAAGGCT	GGTTTCTGGCAAGAACCAACAGCA
AMPK1	CGTGTACGAAGGAAGAATCC	GAGTAGCAGTCCCTGATTTG
PGC1alpha	GAGGGAAAGTGAGCGATTAG	GTGAGGCTGATGTGTACTG
LKB1	GTCACCCTCTACAACATCAC	GTACTCAAGCATCCCTTTCA
beta ACTIN	CGCCGCCAGCTCACCATG	CACGATGGAGGGGAAGACGG

Western blot analysis

After SDS–PAGE, proteins were transferred to Hybond-N Nitrocellulose membrane (GE Healthcare Bio-Sciences). Membranes were blocked in Tris-buffered saline (TBS) containing 5% non-fat dry milk and 0.1% Tween 20 (TBS-T), before incubation with the primary antibody overnight at 4°C. Membranes were then washed with TBS-T followed by exposure to the appropriate horseradish peroxidase-conjugated secondary antibody for 1 hr, and then visualized using the enhanced chemiluminescence (ECL) detection system (GE Healthcare Bio-Sciences) by ProXPRESS 2D (PerkinElmer) scanning. The following antibodies were used: α-enolase mAb clone 72/1 (1), enolase (H300, Santa Cruz Biotechnology), β-actin (A2066, Sigma-Aldrich), c-Myc (GT168, GeneTex), p62/SQSTM1 (N3C1, GeneTex), ATG4B (GeneTex), ATG4D (N3C3, GeneTex), Sirt1 (19A7AB4, Abcam) phospho-Tyr15-cdc2 (9111, Cell Signaling), phospho-Ser807/811-Rb (8516, Cell Signaling), phospho-Ser345-Chk1 (2348, Cell Signaling), phospho-Thr68-Chk2 (2661, Cell Signaling), phospho-Ser15-p53 (9284, Cell Signaling), Cyclin D1 (2926, Cell Signaling), Cyclin D3 (2936, Cell Signaling). LC3-II was enriched and quantified by Western blot analysis using LC3-II Enrichment Kit (Merck Millipore) according to the manufacturer’s instructions.

Trypsin digestion and desalting

Cell pellets were resuspended in 200 μL of 8 M urea, and the protein concentration was measured by the Bradford Assay (BioRad). Proteins were transferred to a 1.5-mL eppendorf tube, reduced by 10 mM dithiothreitol (DTT) for 30 min at 37°C, and then alkylated by 50 mM iodoacetamide for 20 min at room temperature. The concentrated urea in the sample was diluted to a final concentration of 2 M, and the proteins were digested by trypsin at 37°C for 6 hr in a buffer containing ammonium bicarbonate (50 mM, pH 9). The digestion mixture was then acidified by adding glacial acetic acid to a final concentration of 2% and desalted by ZipTip (Millipore).

Tandem mass spectrometry analysis

Peptides were analyzed by highly sensitive reversed-phase liquid chromatography coupled nanospray tandem mass spectrometry (LC-MS/MS) using an LTQ-Orbitrap mass spectrometer (Thermo Fisher) (2). Briefly, the reversed-phase LC column was slurry-packed in-house with 5 μm, 200 Å pore size C_{18} resin (Michrom BioResources) in a 100 μm i.d. × 10 cm long piece of fused silica capillary (Polymicro Technologies) with a laser-pulled tip. After sample injection, the column was washed for 5 min with mobile phase A (0.1% formic acid), and peptides were eluted using a linear gradient of 0% mobile phase B (0.1% formic acid, 80% acetonitrile) to 50% B in 160 min at 200 nL/min, then to 100% B in an additional 10 min for proteomics analysis. The LTQ-Orbitrap mass spectrometer was operated in a data-dependent mode in which each full MS scan (60,000 resolving power) was followed by eight MS/MS scans where the eight most abundant molecular ions were dynamically selected and fragmented by collision-induced dissociation (CID) using a normalized collision energy of 35%. The Dynamic Exclusion Time was 30 s, and the Dynamic Exclusion Size was 200. The “FT master scan preview mode”, “Charge state screening”, “Monoisotopic precursor selection”, and “Charge state rejection” were enabled so that only the 1^+, 2^+, and 3^+ ions were selected and fragmented by CID.

Mass spectrometry data analysis

Tandem mass spectra collected by Xcalibur (version 2.0.2) were searched against the NCBI human protein database using SEQUEST (Bioworks software from ThermoFisher, version 3.3.1) with full tryptic cleavage constraints, static cysteine alkylation by iodoacetamide, and variable methionine oxidation. Mass tolerance for precursor ions was 5 ppm and mass tolerance for fragment ions was 0.25 Da. The SEQUEST search results of proteomics data were filtered by the criteria “Xcorr versus charge 1.9, 2.2, 3.0 for 1^+, 2^+, 3^+ ions; $\Delta Cn > 0.1$; probability of randomized identification of peptide < 0.01”. Confident peptide identifications were determined using these stringent filter criteria for database match scoring followed by manual evaluation of the results. The "false discovery rate (FDR)" was estimated by searching a combined forward-reversed database as described by Elias (3). The SEQUEST search results were exported to Excel files and compared. Spectra count label-free quantitation analysis was performed applying the formula: Relative difference (%) = [(Total no. peptides in shENO1)–(Total no. peptides in shCTRL)]/[(Total no. peptides in shENO1 + Total no. peptides in shCTRL)/2]*100.

Glucose uptake

The uptake of glucose was measured by radiolabeling cells with 0.3 μCi/ml 2-deoxy-D-[^{3}H]-glucose, as described earlier (4) insulin increases cyclic GMP production by inducing nitric oxide (NO). The non carrier-mediated glucose uptake was measuring by performing the assay in the presence of 10 μM cytochalasin B, a strong inhibitor of the facilitated glucose uptake. Results were expressed as pmol 2-deoxy-D-[^{3}H]-glucose/mg cell proteins.

Lactate

Analysis of lactate level was performed on 2×10^6 cells with the L-Lactate Assay Kit (Abcam, Cambridge, MA, USA), following the manufacturer’s instructions. Results were expressed as pmol/10^6cells.

Aldose reductase activity

Cells were washed with PBS, detached by gentle scraping, centrifuged at 13,000 × g for 5 min at 4°C, re-suspended in 0.4 mL of 50 mmol/L Na_3PO_4 buffer (pH 7.0). A 50 µL aliquot was sonicated and used for determining the cell proteins. The remaining sample was transferred to a 96-well plate, in the presence of 10 mmol/L glucose, dissolved in Na_3PO_4 buffer. 1.5 mmol/L NADPH was added. The rate of NADPH oxidation was followed for 6 min, monitoring the absorbance at 340 nm with a Packard microplate reader EL340 (Bio-Tek Instruments). Results were expressed as nmol $NADP^+$ produced/min/mg cell proteins.

NADPH oxidase activity

NADPH oxidase activity was carried out *in vitro* by the isoluminol-chemiluminescence assay. A total of 2×10^6 cells was washed with PBS, detached with trypsin/EDTA (0.05/0.02% v/v), re-suspended in 1 mL PBS containing 7.5 mmol/L glucose, 0.9 mmol/L $CaCl_2$, 0.5 mM $MgCl_2$, and incubated for 10 min at 37°C, in the presence of 200 ng/mL phorbol myristate acetate (PMA) to activate the enzyme, 125 µmol/L isoluminol, 25 U/mL horseradish peroxidase. A 50 µL aliquot was sonicated and used for determining the cell proteins. Chemiluminescence of each sample derived from superoxide and lucigenin was detected using a Synergy HT microplate reader (Bio-Tek Instruments) and was expressed as relative luminescence unit (RLU)/ mg cell proteins. For the negative control, in each experiment, one sample was treated with 450 U/mL superoxide dismutase (SOD): the detected chemiluminescence was < 5% of the corresponding sample in the absence of SOD (not shown).

Tyrosine catabolism

Determination of phenylalanine and acetoacetate levels was performed according to the instruction manuals of EnzyChrom TM phenylalanine and ketone body assay kits (BioAssay Systems).

Glutamine catabolism

Glutamine catabolism was measured as reported (5), with minor modifications. Cells were washed with PBS, detached by gentle scraping, centrifuged at 13,000 × g for 5 min at 4°C, re-suspended in 250 µL of buffer A (150 mmol/L KH_2PO_4, 63 mmol/L Tris/HCl, 0.25 mmol/L EDTA; pH 8.6) and sonicated. The intracellular protein content was measured using the BCA kit (Sigma-Aldrich). A volume of 100 µL of the whole cell lysates was incubated for 30 min at 37°C in a quartz cuvette, in the presence of 50 µL of 20 mmol/L L-glutamine and 850 µL of buffer B (80 mmol/L Tris/HCl, 20 mmol/L NAD^+, 20 mmol/L ADP, 3% v/v H_2O_2; pH 9.4). The absorbance of NADH was monitored at 340 nm using a Lambda 3 spectrophotometer (PerkinElmer). The kinetics was linear throughout the assay. The results were expressed as µmol NADH/min/mg cell proteins, and were considered as an index of the activity of glutaminase (GLS) plus L-glutamic dehydrogenase. In a second series of samples, 20 µL of the GLS inhibitor bis-2-(5-phenylacetamido-1,3,4-thiadiazol-2-yl)ethyl sulfide BTPES (30 µmol/L) was added after 15 min. This concentration was chosen as it produced 100% inhibition of glutaminase activity in our system (not shown). The absorbance of NADH was monitored for 15 min as described previously. The results, considered as an index of the activity of L-glutamic dehydrogenase, were expressed as µmol NADH/min/mg cell proteins. GLS activity was obtained by subtracting the rate of the second assay from the rate of the first one.

Purine synthesis

The activity of glutamine amidophosphoribosyltransferase (GPAT), considered as an index of the de novo synthesis of purine nucleotides, was measured as described previously (6). Cells were washed twice with PBS, detached with trypsin/EDTA (0.05/0.02% v/v), centrifuged at 13,000 × g for 5 min, re-suspended in 1 mL of 50 mmol/L potassium phosphate buffer (pH 7.4), containing 5 mmol/L dithiothreitol (DTT), C100 µg/mL phenylmethanesulfonylfluoride (PMSF), 2 µg/mL aprotinin, and sonicated. Samples were then centrifuged at 40,000 × g for 20 min at 4°C; supernatants were used for protein quantification and GPAT assay. 0.5 mL of each sample was incubated for 1 hr at 37°C with 5 mmol/L 5-phosphoribosyl 1-pyrophosphate, 5 mmol/L MgCl2, 1 mmol/L DTT, 0.75 µCi [^{14}C]-L-glutamine (200 mCi/mmol, PerkinElmer). The [^{14}C]-L-glutamate generated form the reaction was separated from [^{14}C]-L-glutamine by ion exchange chromatography in a 2 mL column. The radioactivity of the eluate containing [^{14}C]-L-glutamate was counted by liquid scintillation and expressed as pmol glutamate/h/mg cell proteins.

Pyrimidine synthesis

The activity of carbamoyl phosphate synthetase II (CPSII) was measured as an index of the *de novo* synthesis of pyrimidine nucleotides. Cells were washed twice with PBS, re-suspended in 0.5 mL of lysis buffer (20 mmol/L Tris/HCl, 1% v/v Triton X-100, 10% v/v glycerol, 137 mmol/L NaCl, 0.150 mmol/L Na_3VO_4, 0.250 mmol/L PMSF, 5 µg/ml leupeptin; pH 7.5), then centrifuged at 10,000 x g for 10 min at 4°C. A 50 µL aliquot was used for the protein content quantification; the remaining lysate was incubated with 0.5 mL of the assay buffer (87 mmol/L Tris/HCl, 87 mmol/L KCl, 25 mmol/l $MgCl_2$, 10 mmol/L ATP, 3.3 mmol/L-glutamine, 17.5 mmol/L-aspartate, 0.8 mmol/L DTT, 6.5% v/v DMSO, 2.2% v/v glycerol, 4 µCi [^{14}C]-$NaHCO_3$ (54 mCi/mmol, PerkinElmer) for 30 min at 37°C. The reaction was stopped by adding 0.2 mL of 80% w/v trichloroacetic acid. To remove the unincorporated $^{14}CO_2$, the tubes were heated at 85°C for 3 hr; the remaining samples, containing [^{14}C]-carbamoyl aspartate were analyzed by liquid scintillation counting.

Results were expressed as pmol carbamoyl aspartate/min/mg cell proteins.

Measurement of mitochondrial respiratory chain

Cells were washed twice in ice-cold PBS, then lysed in 0.5 mL buffer A (50 mmol/L Tris, 100 mmol/L KCl, 5 mmol/L $MgCl_2$, 1.8 mmol/L ATP, 1 mmol/L EDTA, pH 7.2), supplemented with protease inhibitor cocktail III [100 mmol/L AEBSF, 80 mmol/L aprotinin, 5 mmol/L bestatin, 1.5 mmol/L E-64, 2 mmol/L leupeptin and 1 mmol/L pepstatin (MerckMillipore) 1 mmol/L PMSF, 250 mmol/L NaF. Samples were clarified by centrifuging at 650 × *g* for 3 min at 4°C, and the supernatant was collected and centrifuged at 13,000 x *g* for 5 min at 4°C. The new supernatant was discarded, the pellet containing mitochondria was washed in 0.5 mL buffer A and re-suspended in 0.25 mL buffer B (250 mmol/L sucrose, 15 μmol/L K_2HPO_4, 2 mmol/L $MgCl_2$, 0.5 mmol/L EDTA, 5% w/v bovine serum albumin). A 50 μL aliquot was sonicated and used for the measurement of protein content. The activity of mitochondria respiration complexes was measured according to (7). Results were expressed as nmol NAD^+/min/mg mitochondrial protein for complex I, nmol cyt c reduced/min/mg mitochondrial protein for complexes II-III, nmol cyt c oxidized/min/mg mitochondrial protein for complexes IV.

Enolase inhibitor studies

For enolase inhibitor studies, PhAH lithium salt was custom-synthesized by CAGE chemicals. PhAH was dissolved in PBS as a 50 mM stock and stored at −20°C until use.

Enolase enzymatic activity assay

Enolase activity was measured by coupling the reactions of enolase, pyruvate kinase and lactate dehydrogenase, according to (8). After PhAH treatment, cells were washed with PBS, detached by gentle scraping, centrifuged at 13,000 × g for 5 min at 4°C, re-suspended in 0.25 mL of 100 mmol/L Tris buffer (pH 8.0), 10 mmol/L $MgCl_2$, 100 mmol/L KCl, 1 mmol/L 2-phosphoglyceric acid, 0.4 mmol/L ADP, 6.8 U/mL pyruvate kinase, 9.9 U/mL lactate dehydrogenase. 0.2 mmol/L NADH was added. The rate of NADH oxidation was followed for 6 min, monitoring the absorbance at 340 nm with a Packard microplate reader EL340 (Bio-Tek Instruments). Results were expressed as nmol NAD^+ produced/min/mg cell proteins.

Inhibitors and positive controls for biochemical tests

Zopolrestat: 2.5 μmol/L, 3 hr (pre-incubation) – aldose reductase inhibitor. Apocynin: 10 μmol/L, 3 hr (pre-incubation) – NADPH oxidase inhibitor. DHEA: 250 μmol/L, 3 hr (pre-incubation) – pentose phosphate pathway inhibitor. Rotenone: 100 μmol/L, 3 hr (pre-incubation) – mitochondrial chain inhibitor.

Proliferation assay

Cells were seeded in 6-well plates at a density of 1×10^5 cells/well. After 24 hr of serum starvation, cells were grown in complete medium and then detached, stained with trypan blue, and counted every 24 hr. Results represent at least three independent experiments. For antioxidant treatment, PT45 cells were cultured for 7 days with NAC (5 or 10 mM, Sigma-Aldrich) or with TROLOX-C (100 or 250 μM, Sigma-Aldrich), adding fresh medium daily. Results represent at least three independent experiments.

Cell survival assay

Cell survival was assessed by MTT assay. Cells were seeded in 96-well plate at 2×10^3 cells/well and serum starved for 24 hr. After 48 hr of culture in complete medium, 20 μL of 5 mg/μL MTT solution (Sigma-Aldrich) was added to the medium and incubated at 37°C for a further 4 hr. Medium was removed and the insoluble formazan product was dissolved in 200 μl DMSO (Sigma-Aldrich) for 10 min at room temperature. OD values were measured at 570 nm in an ELISA microtiter plate reader (BioRad). Results represent at least three independent experiments.

Colony forming assay

Briefly, a bottom layer of 2.5 mL culture medium containing 0.9% agarose type VII (Sigma-Aldrich) was initially solidified in a 6-well culture plate. Then, 2 mL of 0.45% agarose solution containing 3×10^4 cells was layered on top of each well. Cells were fed twice a week with complete DMEM and incubated at 37°C for 3 weeks. Colonies were counted by optical microscope. Results represent at least three independent experiments.

REFERENCES

1. Moscato S, Pratesi F, Sabbatini A, Chimenti D, Scavuzzo M, Passantino R, Bombardieri S, Giallongo A, Migliorini P. Surface expression of a glycolytic enzyme, α-enolase, recognized by autoantibodies in connective tissue disorders. Eur J Immunol. 2000; 30:3575–84.
2. Zhou W, Capello M, Fredolini C, Piemonti L, Liotta LA, Novelli F, Petricoin EF. Proteomic Analysis of Pancreatic Ductal Adenocarcinoma Cells Reveals Metabolic Alterations. J Proteome Res. 2011; 10:1944–52.
3. Elias JE, Gygi SP. Target-Decoy Search Strategy for Mass Spectrometry-Based Proteomics. In: Hubbard SJ, Jones AR, editors. Proteome Bioinformatics [Internet]. Humana

Press; 2010 [cited 2014 Jul 29]. p. 55–71. Available from: http://link.springer.com.offcampus.dam.unito.it/protocol/10.1007/978-1-60761-444-9_5

4. Bergandi L, Silvagno F, Russo I, Riganti C, Anfossi G, Aldieri E, Ghigo D, Trovati, M, Bosia A. Insulin Stimulates Glucose Transport Via Nitric Oxide/Cyclic GMP Pathway in Human Vascular Smooth Muscle Cells. Arterioscler Thromb Vasc Biol. 2003; 23:2215–21.
5. Curthoys NP, Weiss RF. Regulation of renal ammoniagenesis. Subcellular localization of rat kidney glutaminase isoenzymes. J Biol Chem. 1974; 249:3261–6.
6. Yamaoka T, Kondo M, Honda S, Iwahana H, Moritani M, Ii S, Yoshimoto K, Itakura M. Amidophosphoribosyltransferase limits the rate of cell growth-linked de novo purine biosynthesis in the presence of constant capacity of salvage purine biosynthesis. J Biol Chem. 1997; 272:17719–25.
7. Wibom R, Hagenfeldt L, von Döbeln U. Measurement of ATP production and respiratory chain enzyme activities in mitochondria isolated from small muscle biopsy samples. Anal Biochem. 2002; 311:139–51.
8. Beutler E. Red Cell Metabolism, a Manual of Biochemical Methods. New York and London: Grune & Stratton Editors; 1975.
